# Supplementary material for: Biomass burning aerosols in most climate models are too absorbing
Source: Nat Commun. 2021 Jan 12;12:277. doi: 10.1038/s41467-020-20482-9 (PMC7804930; doi:10.1038/s41467-020-20482-9)
Supplement: Supplementary file 1 — Supplementary Information [file 41467_2020_20482_MOESM1_ESM.pdf]

Supplementary Information for
Biomass burning aerosols in most climate models are too absorbing

Brown et al.

Supplementary Table 1: Model specifications and additional information. Much of this was taken from the AeroCom Phase III biomass burning (BB) project wiki (https://wiki.met.no/aerocom/phase3-experiments). If different acronyms for the model simulations are used in the paper, these are included in parentheses below the model name.

**Model**	**Resolution**	**Period and Temporal Resolution**	**Nature of the Model**	**Aerosol Species**	**Aerosol Removal**	**Boundary Layer Definition**	**BB Emission Injection Height**	**Aerosol Treatment**	**References**
CAM5.4-MAM4 (CAM5.4)	0.9˚x1.25˚, 30 levels	2003-2011 (monthly)	General Circulation Model (GCM), free-running	Dust, sea salt, black carbon (BC), primary organic aerosol (POA), secondary organic aerosol (SOA), sulfate (SO_4)	Aerosol wet removal is parameterized separately for stratiform and convective clouds. Dry deposition velocities are calculated with model. Gravitational settling.[1]	Diagnostic TKE-based 1st-order K diffusion scheme with entrainment parameterization[2]	Prescribed, ecosystem-specific emission profiles from 0 to 6 km.	4-mode version of the Modal Aerosol Module (MAM4)[3]	Liu et al.[1] Neale et al.[4]

CAM5.4-MAM4_BrC (CAM5.4_BrC and CAM5.4_BrCbl)	0.9˚x1.25˚, 30 levels	2003-2011 (monthly)	General Circulation Model (GCM), free-running	Dust, sea salt, black carbon (BC), primary organic aerosol (POA), secondary organic aerosol (SOA), sulfate (SO_4). Includes brown carbon (BrC) from biomass burning and biofuel sources[5]	Aerosol wet removal is parameterized separately for stratiform and convective clouds. Dry deposition velocities are calculated with model. Gravitational settling.[1]	Diagnostic TKE-based 1st-order K diffusion scheme with entrainment parameterization[2]	Prescribed, ecosystem-specific emission profiles from 0 to 6 km.	4-mode version of the Modal Aerosol Module (MAM4)[3]	Liu et al.[1] Neale et al.[4]
CAM5.3	1.9˚x2.5˚, 30 levels	2008 (daily)	GCM, nudged by ERA-Interim reanalysis horizontal winds	Dust, sea salt, BC, POA, SOA, SO_4	Aerosol wet removal is parameterized separately for stratiform and convective clouds. Dry deposition velocities are calculated with model. Gravitational settling.[1]	Diagnostic TKE-based 1st-order K diffusion scheme with entrainment parameterization[2]	Prescribed, ecosystem-specific emission profiles from 0 to 6 km.	3-mode version of the Modal Aerosol Module (MAM3)[1]	Liu et al.[1] Neale et al.[4]

ECHAM6.3-HAM2.3-SALSA2.0 (ECHAM6.3-SALSA2)	1.9˚x1.9˚, 47 levels	2008 (daily)	GCM, nudged by ERA-Interim reanalysis data	Dust, sea salt, BC, organic aerosol (OA), SO_4	Wet deposition, dry deposition and sedimentation[6,7]	Equation 3[8]	Prescribed, ecosystem-specific emission profiles from 0 to 6 km.	Sectional Aerosol module for Large Scale Applications, version 2 (SALSA2)[9]	Laakso et al.[10] Kokkola et al.[9]
ECHAM6.3-HAM2.3-M7 (ECHAM6.3-HAM2.3)	1.9˚x1.9˚, 31 levels	2008 (daily)	GCM, nudged by ERA-Interim reanalysis data	Dust, sea salt, BC, OA, SO_4	Wet deposition[11,12], dry deposition[13] and sedimentation[14,15]	Equation 3[8]	Injection heights of biomass burning emissions follow the recommendations of Val Martin et al.[16]: 75% of the emissions are evenly distributed within the planetary boundary layer (PBL), 17% in the first level, and 8% in the second level above the PBL	Version 2 of the Hamburg Aerosol Module (HAM2)[13]	Zhang et al.[13] Tegen et al.[17]
GEOS-Chem	4˚x5˚, 10 levels	2005 (monthly)	Off-line Chemical Transport Model (CTM)	Dust, sea salt, BC, OA, SO_4, nitrate (NO_3)	Scavenging by snow and cold/mixed precipitation (Wang et al.[18]). Wet deposition: Liu et al.[19] for water-soluble aerosols. Gravitational settling: Fairlie et al.[20] for dust, Alexander et al.[21] for coarse sea salt.	Recalculated internally as a function of atmospheric stability[22]	Species are emitted in the lowest model level, and mixed homogeneously up to the mixing height (PBL)	Bulk aerosol treatments described in Bey et al.[23] and Saleh et al.[24]	Bey et al.[23] Saleh et al.[24]

HadGEM3	1.25˚x1.875˚, 85 levels*<br><br>*interpolated to 25 levels for this study	2008 (daily)	Atmosphere-only GCM, nudged by ERA-Interim reanalysis data	Dust, sea salt, BC, organic carbon (OC), SO_4	GLOMAP-mode scheme[25]	Diagnosed from stability profile (non-local scheme accounting for moist parcel ascent)	BB emission are evenly distributed within the lowest 3km of the troposphere.	The modal Global Model for Aerosol Processes (GLOMAP-mode)[25]	Bellouin et al.[26]; Johnson et al.[27]
OsloCTM2	2.8˚x2.8˚, 60 levels	2008 (monthly)	Off-line CTM	Dust, sea salt, BC, POA, SOA, SO_4, NO_3	Dry deposition: velocity for each component is dependent on season and surface type. Wet deposition: Soluble components are removed proportionally to the fraction of the clouds which rains out.	PBL-height given in the IFS data.	Biomass burning emission injection height from project RETRO, between 0 and 5 km[28]	Bulk scheme for carbonaceous aerosols with aging dependent on season and latitude. Aerosol treatments described in Skeie et al.[29]	Myhre et al.[30]; Skeie et al. [29]

Supplementary Table 2: Model treatments of biomass aerosol properties affecting biomass burning smoke single-scattering albedo. If different acronyms for the model simulations are used in the paper, they are included in parenthesis below the model name.

Model	BC Refractive Index (at λ = 550 nm)*	Brown Carbon	OM:OC	Mixing State	Dry Number Diameter (D_g)	Sigma	BB emission dataset	Aerosol Hygroscopicity
CAM5.4-MAM4 (CAM5.4)	1.95+0.79i [31]	None	1.4 [1]	Internally mixed (volume mixing treatment) BC and POA in a primary carbon mode that can age into an external, internally mixed accumulation mode[1]	Primary Carbon mode: 39 – 130 nm<br><br>Accumulation mode: 58 – 270 nm [1]	Primary Carbon mode: 1.6<br><br>Accumulation mode: 1.8 [1]	GFED v3.1 (daily)	BC: κ = 0<br>POA: κ = 0.1 [1]
CAM5.4-MAM4_BrC (CAM5.4_BrC and CAM5.4_BrCbl)	1.95+0.79i [31]	Brown carbon from biomass burning and biofuel sources[5] following Saleh et al.[32] and including a photochemical bleaching option based on Wang et al.[33]	1.4 [1]	Internally mixed (volume mixing treatment) BC and POA in a primary carbon mode that can age into an external, internally mixed accumulation mode[1]	Primary Carbon mode: 39 – 130 nm<br><br>Accumulation mode: 58 – 270 nm [1]	Primary Carbon mode: 1.6<br><br>Accumulation mode: 1.8 [1]	GFED v3.1 (daily)	BC: κ = 0<br>POA: κ = 0 [34]
CAM5.3	1.95+0.79i [31]	None	1.4 [1]	Internally-mixed (volume mixing treatment) accumulation	Accumulation mode: 58 - 270 nm [1]	1.8 [1]	GFED v3 (daily)	BC: κ = 0<br>POA: κ = 0.1 [1]

				mode[1]				
ECHAM6.3-HAM2.3-SALSA2.0 (ECHAM6.3-SALSA2)	1.85+0.71i [31]	None	1.4 [7]	Internally mixed (volume mixing treatment) in separate insoluble and soluble subclasses[9]	150 nm [7]	Bin Scheme[9]	GFED v3 (daily)	Use parameterizations by Jacobson[35] to infer hydration from binary molalities of inorganic salts[6]
ECHAM6.3-HAM2.3-M7 (ECHAM6.3-HAM2.3)	1.85+0.71i [31]	None	1.4 [7]	Internally Mixed (volume mixing treatment) within externally mixed soluble and insoluble modes[13]	Insoluble/soluble Aiken modes:<br>60 nm<br><br>Soluble Accumulation mode:<br>150 nm [13]	1.59 [7]	GFED v3 (daily)	BC: κ = 0<br>POA: κ = 0.06 [13]
GEOS-Chem	1.85+0.71i [31]	No brown carbon **or** brown carbon parameterized following Saleh et al.[32] and Saleh et al.[24]	2.1 [36]	Externally mixed **or** internally mixed (core-shell treatment) BC, POA, SO_4, NO_3 [24]	Externally Mixed:<br>BC: 150 nm [37]<br><br>POA: 200 nm [24]<br><br>Internally mixed:<br>Size depends on volume fraction of BC coating[24]	1.59 [37]	GFED v3 (monthly)	BC: κ = 0<br>POA: κ = 0.14 [38]
HadGEM3	1.75+0.44i [39]	None	1.4 [27]	Internally mixed (volume mixing treatment) and BB initially in insoluble modes ages into soluble modes by condensation of volatile compounds [27]	150 nm [7]	1.59 [7]	GFED v3 (daily)	Once aged, OC takes up 25% of water taken up by equivalent mass of H_2SO_4 [27]
OsloCTM2	BB:<br>1.54+0.018i [40]	None	2.6 (BB) [41]	Internally mixed (volume mixing	Mode 1: 240 nm<br>(num. fraction = 0.996)	Mode 1: 1.3<br><br>Mode 2: 1.5 [40]	GFED v3 (daily)	Water uptake based on Magi and Hobbs[43] scattering

				treatment) BC and OC in a three-mode BB treatment[42]	Mode 2: 520 nm (num. fraction = 0.033) [40]			growth factor for aged heavy smoke[30]

*All RIs are for BC except for OsloCTM2, which is for biomass burning aerosol

Supplementary Table 3: Comparison of accumulation mode (Mode 1) and primary carbon mode (Mode 4) from biomass burning (BB) regions in the lowest 3 levels of the CAM5.4. Simulations can be found in Supplementary Table 6. The aerosol properties analyzed are total number concentration (N_t), volume extinction coefficient (β_{ext}), and single scattering albedo (SSA). For information regarding the modes used in the 4-Mode version of the modal aerosol model (MAM4), see Liu et al.[3]. Modes 2 and 3 (Aitken and coarse, respectively) have a very small contribution in these BB regions, and so are excluded in this comparison.

**Model Simulations**	**N_t (cm^{-3})**		**β_{ext} (m^{-1})**		**SSA**	
	**Mode 1**	**Mode 4**	**Mode 1**	**Mode 4**	**Mode 1**	**Mode 4**
North America						
CAM5.4	465	4,680	0.2×10^{-4}	0.4×10^{-4}	0.902	0.821
CAM_Dg	482	4,387	0.2×10^{-4}	0.5×10^{-4}	0.901	0.843
CAM_Dg160	240	1,519	0.2×10^{-4}	0.6×10^{-4}	0.904	0.860
Africa						
CAM5.4	1,817	14,004	0.8×10^{-4}	1.1×10^{-4}	0.833	0.732
CAM_Dg	1,847	13,118	0.8×10^{-4}	1.2×10^{-4}	0.832	0.752
CAM_Dg160	1,034	4,776	0.8×10^{-4}	1.5×10^{-4}	0.838	0.786
South America						
CAM5.4	2,165	20,223	1.8×10^{-4}	2.1×10^{-4}	0.835	0.72
CAM_Dg	2,233	18,265	1.9×10^{-4}	2.4×10^{-4}	0.835	0.755
CAM_Dg160	1,300	7,128	1.7×10^{-4}	3.0×10^{-4}	0.843	0.785

Supplementary Table 4: Observation details. Includes instruments used for the necessary data in this study. Latitude and longitude correspond to the general location of the biomass burning observations. The heading acronym BC/EC indicates black carbon/elemental carbon, and OA (+ IA) indicates organic aerosol – with some studies including inorganic aerosol. Undefined instrument acronyms are referenced in the Methods section.

**Dataset**	**Date(s)** ***Time**	**Latitude (N)**	**Longitude (E)**	**BC/EC**	**OA (+ IA)**	**Abs. Coeff.**	**Scat. Coeff. OR Ext. Coeff. (♦)**	**SSA wavelength (nm)**	**Number Conc.**	**Conditions**	**Reference**
ARCTAS_2008	20080701	54.46˚	-110.17˚	SP2	AMS	PSAP	NEPH	532, 660	UHSAS (78-986 nm)	Flight through Saskatchewan, Canada biomass burning plume. Multiple passes were made at a height of ~600 m above ground from 0 to 110 km from the fire. Estimated age of sampled smoke ranges from minutes to 2-3 days.	Jacob et al.[44] https://cloud1.arc.nasa.gov/arctas/docs/flight/2008-7-1_dc8_report.pdf
Artaxo_2013	200802 - 201206	-8.69˚ (PVH) -2.59˚ (TT34)	-63.87˚ (PVH) -60.21˚ (TT34)	–	–	–	–	–	TSI Condensation Particle Counter (CPC) (10-500 nm)	Long-term analysis of biomass smoke influence during the dry season at a relatively pristine, Central Amazon site (TT34; dry season: July-December) and a southwestern Amazon site exposed to slash and burn biomass emissions near	Artaxo et al.[45]

										Porto Velho (PVH; dry season: June-December). The range in D_g is representing biomass aerosol differences between the two sites.	
Brito_2014	20120913-20120930	-8.69˚	-63.87˚	–	–	–	–	–	SMPS (10-430 nm)	Observations from a site 4 km north of Puerto Velho, Brazil during the SAMMBA field experiment. Range in D_g reflects a transition from fresh to aged biomass smoke.	Brito et al.[46]
DC3_2012	20120622<br><br>20:45 – 20:52 UTC	41.02˚	-104.31˚	SP2	AMS	PSAP	NEPH	550, 700	UHSAS (63-1000 nm)	Flight during the DC3 field campaign, in which a ~1.5 km thick smoke layer – centered at a ~6 km atltitude – was sampled along the Colorado-Wyoming border. The sampled smoke originated from the High Park Fire in Northern Colorado and was aged ~2 hours.	Barth et al.[47]<br><br>http://catalog.eol.ucar.edu/dc3_2012/missions/missions.html
GoAma	201409	-3.21˚	60.6˚	SP2	ACSM	PSAP	NEPH	550, 700	UHSAS	Observations	Martin et

zon_2014	30-20141001								(60-1000 nm)	from the "T3" site located approximately 70 km downwind of Manaus, Brazil during the GoAmazon campaign. The two days used in this comparison are during the dry season, with both days showing strong regional biomass burning influence. Aerosol influence assumed to range from fresh to aged (1-2 days).	al.[48]<br><br>de Sá et al.[49]
Grieshop_2017	201109-201208	15.62˚	76.3˚	Thermal optical analysis following NIOSH 5040 method	Thermal optical analysis following NIOSH 5040 method	PSAP	Portable emission measurement system (PEMS)	660	–	Emission analysis of low and high efficiency cook stoves in Hire Waddarkal in the Koppal district of Karnataka State, India	Grieshop et al.[50]
Haywood_2003	20020905-20020916	-22.56˚	17.07˚	–	–	–	–	–	Particle Measuring System (PMS) Passive Cavity Aerosol Spectrometer Probe (PCASP) (50-1500 nm)	Flights from Windhoek, Namibia during the SAFARI 2000 campaign sampling agricultural burns near Otavi, Namibia (fresh smoke), as well as plumes transported over the ocean and	Haywood et al.[40]

										along the Namibian and Angola coasts (aged smoke). Range in D_g reflects a difference between fresh and aged biomass smoke.	
Liu_2014	201210-201211	–	–	Filter and thermal optical transmittance test	Filter and thermal optical transmittance test	PASS-3	PASS-3	532, 781	–	Controlled burns for the FLAME-4 lab experiment. Fresh smoke emissions from 20 unique fuels from a variety of different geographic locations contribute to these data.	Liu et al.[51]
Martins_1998	19950916-19950917	-5.37˚	-49.15˚	–	–	–	–	–	PCASP (100-3000 nm) and DMPS (10-600 nm)	Aircraft sampling of biomass burning plumes near Marabá, Brazil during the SCAR-B campaign. Range in D_g is representing differences in flaming emissions (from trees, brush, grass, and pasture) and smoldering emissions (from primary forest, undergrowth, and	Martins et al.[52]<br><br>Hobbs[53]

										pasture).	
ORACLES_2016	20160902<br><br>9:18 – 10:26 UTC	-16˚	9˚	SP2	AMS	PSAP	NEPH	530, 660	UHSAS (60-1000 nm)	Flight during the ORACLES campaign through above-cloud biomass burning aerosol off the Angola coast. This aerosol pass ranged from about 2-5 km above sea level. Based on HYSPLIT trajectory analysis, the age of smoke in this pass likely ranged from ~1 day to >6 days.	Zuidema et al.[54]<br><br>https://espo.nasa.gov/oracles/science_reports/ORACLES_-_P-3_Orion_09_02_16_Science_Report
Pokhrel_2016	20121115-20121116	–	–	Filter and thermal optical transmittance test	Filter and thermal optical transmittance test	Potoacoustic absorption spectrometer (PAS)	Cavity ring-down spectrometer (CRDS) (♦)	532, 660	–	Controlled burns for the FLAME-4 lab experiment. 12 unique fuels from a variety of different geographic locations contribute to these data. Smoke age ranges from 15 min to several hours.	Pokhrel et al.[55]
SEAC^{4}RS_BW_2013	20130806<br><br>19:24 – 22:34 UTC	41.8˚	-124.02˚	SP2	AMS	PSAP	NEPH	550, 700	UHSAS (63-891 nm)	Flight during the SEAC^{4}RS campaign sampling above-cloud biomass burning emissions near Crescent City,	Toon et al.[56]<br><br>https://espo.nasa.gov/seac4rs/flight_reports/DC-

										CA from the Big Windy Fire complex in Oregon. These passes ranged from 2-3 km over stratus, to 300-600 meters over the Oregon coastal range. Smoke age ranges from hours to ~1 day.	8_08_06_13_-_08_07_13
SEAC^{4}RS_RF_2013	20130826<br><br>23:02 – 23:18 UTC	39.56˚	-119.43˚	SP2	AMS	PSAP	NEPH	550, 700	SMPS (11-316 nm)	Flight during the SEAC^{4}RS campaign sampling near-source emissions from the Rim Fire in California. This plume was sampled from ~5-4 km MSL near Reno, Nevada. Smoke samples represent the first ~6 hours of aging.	Mason et al.[57]<br><br>Toon et al.[56]<br><br>https://espo.nasa.gov/seac4rs/flight_reports/DC-8_08_26_13_-_08_27_13
Stockwell_2016	20150905-20150906	-2.44˚	114.17˚	Calculated from recommended BC mass absorption coefficient (MAC) of 4.74 m^2g^{-1}	Thermal optical analysis following NIOSH 5040 method	Measured directly at 1 s time resolution using two PAXs (Droplet Measurement Technologies, Inc., CO)		870	–	–	Stockwell et al.[58]

				and measured absorption coefficient							
UWMLCA_2017	20171013-20171017	37.57˚	-121.14˚	Calculated from assumed BC mass absorption coefficient (MAC) of 6.25 ± 1 m^2g^{-1} and measured absorption coefficient	SMPS total volume and AMS density	UWPAS	CAPS	660	–	University of Wyoming mobile lab measurements of the Tubbs and Lion fires in California. Smoke ages range from approximately 6 hours to ~1 day.	Foster et al.[59]
UWMLMT_2017	20170827-20170829	46.98 ˚	-113.05˚	Calculated from assumed BC mass absorption coefficient (MAC) of 6.25 ± 1 m^2g^{-1} and measured	SMPS total volume and AMS density	University of Wyoming Photoacoustic Spectrometer (UWPAS)	Cavity attenuated phaseshift spectrometer (CAPS)	660 nm	–	University of Wyoming mobile lab measurements of the Rice Ridge fire in Montana. Two measurements sites were closer to the fire and one was further downwind. Some possible contribution from the Lolo Peak fire. Smoke ages	Foster et al.[59]

				absorption coefficient						range from ~ 6-12 hours.	
Welgegund_2011	20100901-20110816	-26.57˚	26.54	MAAP	ACSM	MAAP	NEPH	637	DMPS (12-840 nm)	Site located approximately 100 km west of Johannesburg, South Africa. Biomass burning plumes originated from either grasslands, croplands, or savannah. DMPS data is from Vakkari et al.[60] and represents a range in aerosol age from fresh (<0.5 hr) to aged (2.5-3.5 hr).	Vakkari et al.[60] Vakkari et al.[61]
Yokelson_2009	20060323	19.65˚	-89.3˚	Assumptions based on measured ΔBC / $\Delta PM_{2.5}$. See Pokhrel et al.[55]		PSAP	NEPH	532	–	Along track sampling of biomass plume in Mexico during the MILAGRO project. Smoke sampled represents the first ~1.5 hours of aging.	Yokelson et al.[62] Pokhrel et al.[55]

*Times are included only when a section of the sampling flight is used for biomass burning comparison.

Supplementary Table 5: Observational data processing constraints and uncertainty bounds for black carbon (BC), organic aerosol (OA), single scattering albedo (SSA), and mass absorption cross-section (MAC) data

Dataset	OM:OC	BC Unc.	BC Conc. Threshold (μg m^{-3})	OA Unc.	BC/(BC+OC) Unc.	Abs. Coeff. Unc.	Scat. Coeff. Unc.	SSA Unc.	MAC Unc.
ARCTAS	Assumed 2.0	30% (from datafile)	0.9	38% (from datafile)	Calculated with Gaussian error propagation	20%	–	5% and data corrected following Virkkula et al.[64] (from datafile)	Calculated with Gaussian error propagation
DC3	Measured	30% (from datafile)	0.9	38% (from datafile)	Calculated with Gaussian error propagation	20% [63]	–	5% (from datafile)	Calculated with Gaussian error propagation
GoAmazon	Assumed 2.0	30% (from datafile)	0.3	38% (from datafile)	Calculated with Gaussian error propagation	20% [63]	10% [64]	Calculated with Gaussian error propagation	Calculated with Gaussian error propagation
ORACLES	Calculated from measured O:C based on Aiken et al.[66]	30% (from datafile)	0.9	50% (from datafile)	Calculated with Gaussian error propagation	20% [63]	10% [65]	Calculated with Gaussian error propagation	Calculated with Gaussian error propagation
SEAC[4]RS_BW	Measured	30% (from datafile)	0.9	38% (from datafile)	Calculated with Gaussian error propagation	5% (from datafile)	5% (from datafile)	5% (from datafile)	Calculated with Gaussian error propagation
SEAC[4]RS_RF	Measured	30% (from datafile)	0.9	38% (from datafile)	Calculated with Gaussian error propagation	5% (from datafile)	5% (from datafile)	5% (from datafile)	Calculated with Gaussian error propagation
UWML	Assumed 1.5	6% (absorption coefficient) 16% (BC mass absorption coefficient, assumed 6.25 $m^2 g^{-1}$)	–	20% (SMPS total aerosol volume) 38% (AMS density, assumed 1.5 g cm^3)	Calculated with Gaussian error propagation	–	–	6% [59]	16%
Welgegund	Assumed 2.0	12% [67]	0.3	20% [68]	Calculated with Gaussian error propagation	12% [67]	10% [68]	Calculated with Gaussian error propagation	–
Yokelson_2009	–	–	–	–	Assumed 40%	–	–	Assumed 5% (based	–

					(based on average observational uncertainties)			on average observational uncertainties)	

Supplementary Table 6: CAM5.4 model simulations used in this study.

Model Run	Simulation type	Ensembles	Description
CAM5.4	Free-running	5	Default CAM5.4
CAM_NOBB	Free-running	5	CAM5.4 without emissions from biomass burning BC, POA, SO_4, and SO_2.
CAM_BCRI	Free-running	5	CAM5.4 with a lowered BC refractive index based on Bond and Bergstrom[31]
CAM_DG	Free-running	5	CAM5.4 with an expanded Mode 4 mean diameter range, changed from 10-100 nm to 10-300 nm.
CAM_DG160	Free-running	5	CAM_DG with Mode 4 aerosol geometric mean diameter increased (by decreasing Mode 4 number emissions) to ~160 nm
CAM_EMIX	Free-running	5	CAM5.4 with an externally mixed Mode 4 biomass burning aerosol
CAM_ALL	Free-running	5	CAM5.4 with the modifications from CAM_BCRI, CAM_DG160, and CAM_EMIX.

Supplementary Table 7: Dry geometric mean diameter (D_g), geometric standard deviation (σ_g), and total number concentration (N_t) for the primary carbon mode (Mode 4) within the CAM5.4 biomass burning regions. These regions are identified in Supplementary Figure 1. The different model simulations are default (CAM5.4), default with a broadened geometric mean diameter (D_g) range (CAM_Dg), and the default with the broadened D_g range and decreased number emissions to better match the observational D_g of 160.1 nm (CAM_DG160). For observational data, see Supplementary Table 8.

**Model Simulations**	**D_g (nm)**	**σ_g**	**N_t (cm^{-3})**
North America			
CAM5.4	90.8	1.6	4,680
CAM_DG	106.1	1.6	4,387
CAM_DG160	146.6	1.6	1,519
Africa			
CAM5.4	100	1.6	14,004
CAM_DG	112.5	1.6	13,118
CAM_DG160	161.2	1.6	4,776
South America			
CAM5.4	99.9	1.6	20,223
CAM_DG	119.6	1.6	18,265
CAM_DG160	170.0	1.6	7,127
Global Average			
CAM5.4	96.9		
CAM_DG	112.7		
CAM_DG160	159.1		

Supplementary Table 8: Geometric mean diameter (D_g), geometric standard deviation (σ_g), and total number concentration (N_t) for biomass burning (BB) observations.

**Observations**	**D_g (nm)**	**σ_g**	**N_t (cm^{-3})**
North America			
$SEAC^4RS$_BW	281.8	1.43	124,441
$SEAC^4RS$_RF	177.8	1.26	1,082,390
DC3	226.5	1.48	148,719
ARCTAS	143	1.51	501,283
Africa			
ORACLES	164.5	1.45	397,104
Welgegund	96 (69–124)	1.62 (1.71–1.52)	519 (761–277)
Haywood et al.[40]	220 (200–240)	1.3	16,012 (1066–30,957)
South America			
GoAmazon	116.1	1.53	4,172
Brtito et al.[46]	120 (110–130)	–	–
Artaxo et al.[45]	110 (90–130)	–	–
Martins et al.[52]	105.7 (87–120)	1.88 (1.87–1.89)	–
Mean =	160.1	1.50	

Supplementary Table 9: Equations for the linear regression for observations and models from the biomass burning (BB) single scattering albedo at 550 nm wavelength (SSA_{550}) versus black carbon to total carbon (BC:TC) comparison. ±1 standard deviation are included for the intercept and slope of the observations. Inter-annual variation in y-intercept and slope are included for the multi-year CAM5.4 simulations – CAM5.4, CAM5.4_BrC, and CAM5.4_BrC (w/bleaching) – used in Fig. 2.

**Source**	**Linear Regression Equation**
Observations	SSA_{550} = 0.969±0.002 - 0.779±0.017 × BC:TC
CAM5.4	SSA_{550} = 0.971(0.914 – 0.998) -1.879(-1.285 – -2.749) × BC:TC
CAM_BCRI	SSA_{550} = 0.980 -1.713 × BC:TC
CAM_DG160	SSA_{550} = 0.975 -1.624 × BC:TC
CAM_EMIX	SSA_{550} = 0.991 -1.546 × BC:TC
CAM_AI	SSA_{550} = 0.996 -1.211 × BC:TC
CAM5.4_BrC	SSA_{550} = 0.869(0.835 – 1.023) -1.980(-1.261 – -4.689) × BC:TC
CAM5.4_BrC (bleaching)	SSA_{550} = 0.849(0.823 – 1.056) -1.593(-1.003 – -5.225) × BC:TC
CAM5.3	SSA_{550} = 0.979 -1.913 × BC:TC
ECHAM6.3_SALSA2	SSA_{550} = 0.945 -1.438 × BC:TC
ECHAM6.3_HAM2.3	SSA_{550} = 0.997 -2.169 × BC:TC
HadGEM3	SSA_{550} = 1.001 -1.477 × BC:TC
OsloCTM2	SSA_{550} = 1.000 -1.173 × BC:TC
GEOS-Chem	
NA + EM	SSA_{550} = 0.972 -0.625 × BC:TC
A + EM	SSA_{550} = 0.957 -1.033 × BC:TC
NA + IM	SSA_{550} = 0.899 -0.975 × BC:TC
A + IM	SSA_{550} = 0.886 -1.191 × BC:TC

Supplementary Table 10: Global and regional averages of biomass burning (BB) single scattering albedo (SSA) for CAM5.4 and the BB microphysics sensitivity simulations used in this study. Averages for SSA from North Asia and Southeast Asia regions are neglected in observations due to the unavailability of 550 nm SSA data. The percentage change in CAM5.4 to reach the modified simulation value is reported in parentheses.

Source	Global Avg. SSA	Africa	South America	North America	North Asia	Southeast Asia
Observations	0.92	0.846	0.917	0.951	–	–
CAM5.4	0.859	0.778	0.774	0.863	0.858	0.778
CAM_BCRI	0.877 (+2.1%)	0.803 (+3.3%)	0.8 (+3.4%)	0.88 (+2.0%)	0.876 (+2.1%)	0.797 (+2.4%)
CAM_DG160	0.878 (+2.1%)	0.806 (+3.6%)	0.804 (+3.8%)	0.881 (+2.1%)	0.88 (+2.5%)	0.819 (+5.3%)
CAM_EMIX	0.899 (+4.6%)	0.824 (+6.0%)	0.823 (+6.3%)	0.901 (+4.4%)	0.899 (+4.7%)	0.841 (+8.1%)
CAM_ALL	0.923 (+7.4%)	0.868 (+11.6%)	0.869 (+12.3%)	0.927 (+7.4%)	0.928 (+8.1%)	0.889 (+13.9%)

Supplementary Table 11: Global and regional averages of biomass burning (BB) absorption relative to extinction (1-SSA) for CAM5.4 and the BB microphysics sensitivity simulations used in this study. Averages for 1-SSA from North Asia and Southeast Asia regions are neglected in observations due to the unavailability of 550 nm SSA data. The percentage change in CAM5.4 to reach the modified simulation value is reported in parentheses.

Source	Global Avg. 1-SSA	Africa	South America	North America	North Asia	Southeast Asia
Observations	0.08	0.154	0.083	0.049	–	–
CAM5.4	0.141	0.222	0.226	0.137	0.142	0.222
CAM_BCRI	0.123 (-12.8%)	0.197 (-11.3%)	0.2 (-11.5%)	0.12 (-12.4%)	0.124 (-12.7%)	0.203 (-8.6%)
CAM_DG160	0.122 (-13.5%)	0.194 (-12.6%)	0.196 (-13.3%)	0.119 (-13.1%)	0.12 (-15.5%)	0.181 (-18.4%)
CAM_EMIX	0.101 (-28.4%)	0.176 (-20.7%)	0.177 (-21.7%)	0.099 (-27.7%)	0.099 (-30.3%)	0.159 (-28.4%)
CAM_ALL	0.077 (-45.4%)	0.132 (-40.5%)	0.131 (-42.0%)	0.073 (-46.7%)	0.072 (-49.3%)	0.111 (-50.0%)

Supplementary Table 12: CAM5.4 Mode 4 biomass burning primary organic aerosol and black carbon lifetimes (days) calculated over global, Tropical (25˚S – 25˚N), and Arctic (60˚N – 90˚N) averages.

Source	Global	Tropics (25˚S – 25˚N)	Arctic (60˚N – 90˚N)
CAM5.4			
POA BB Lifetime	2.77	2.8	3.06
BC BB Lifetime	2.78	2.82	3.09
CAM_BCRI			
POA BB Lifetime	2.76	2.79	3.09
BC BB Lifetime	2.76	2.79	3.11
CAM_DG160			
POA BB Lifetime	2.62	2.74	2.37
BC BB Lifetime	2.65	2.74	2.39
CAM_EMIX			
POA BB Lifetime	2.75	2.78	3.10
BC BB Lifetime	2.75	2.79	3.12
CAM_ALL			
POA BB Lifetime	2.57	2.7	2.31
BC BB Lifetime	2.6	2.7	2.32

Supplementary Table 13: Comparison of globally averaged biomass burning (BB) radiative effect due to aerosol-radiation interactions (RE_{ari}) from CAM5.4, ECHAM6.3-HAM2.3, and GEOS-Chem simulations (Saleh et al.[24]). The CAM5.4 simulation RE_{ari} is calculated for specific years to allow comparison to the single-year GEOS-Chem and ECHAM6.3-HAM2.3 simulations.

Model Simulation	Simulation Year	BB RE_{ari} (W m^{-2})
CAM5.4	2005	0.043
CAM5.4_BrC	2005	0.268
CAM5.4_BrCbl	2005	0.215
CAM_ALL	2005	-0.040
GEOS-Chem	2005	
NA+EM		-0.460
A+EM		-0.240
NA+IM		-0.070
A+IM		0.050
CAM5.4	2008	0.042
CAM5.4_BrC	2008	0.269
CAM5.4_BrCbl	2008	0.200
CAM_ALL	2008	-0.022
ECHAM6.3-HAM2.3	2008	0.082

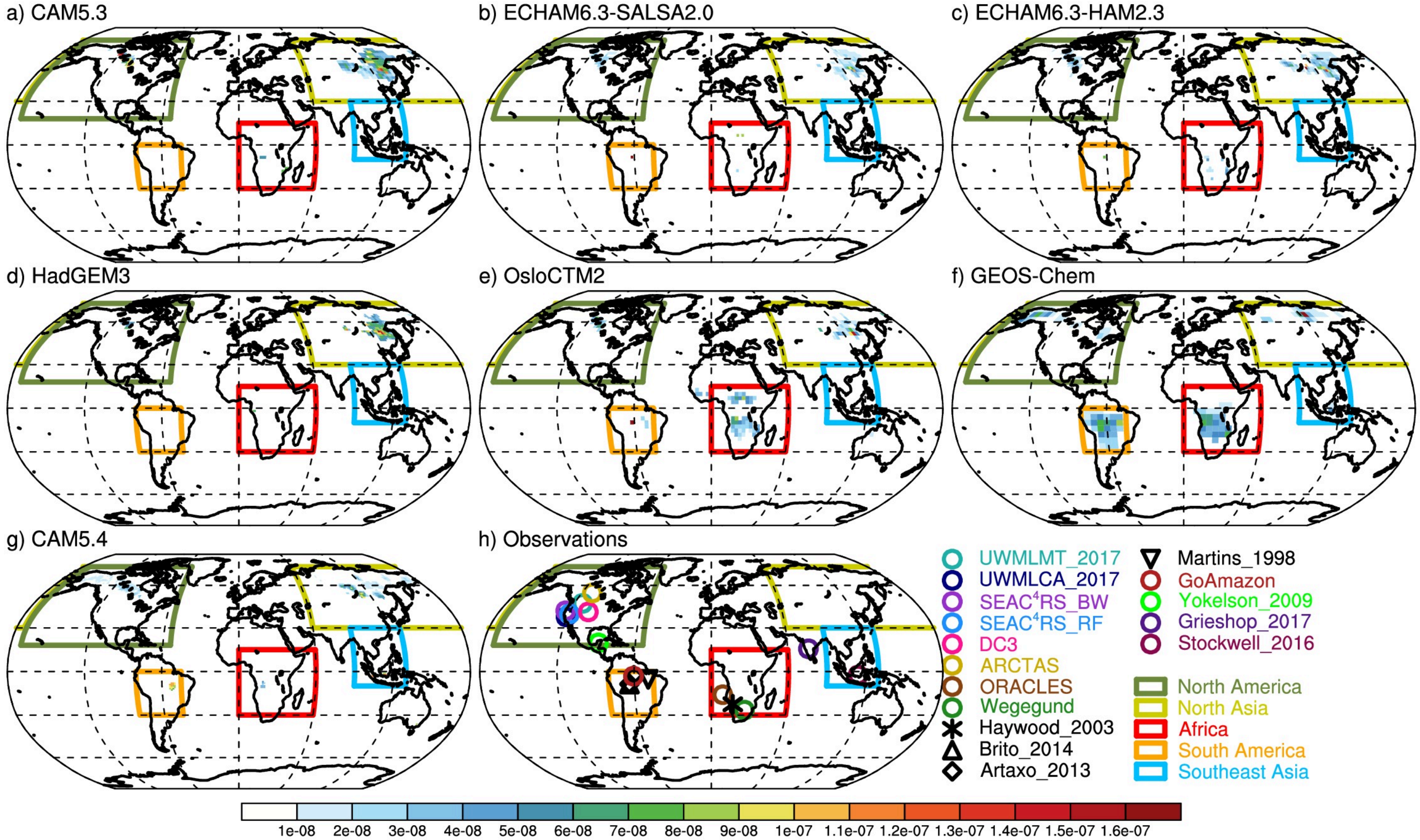

Supplementary Figure 1: Model BB aerosol mass mixing ratio (kg kg^{-1}) of black carbon (BC) + primary organic aerosol (POA) from the lowest level in regions dominated by BB (panels a-g). These panels correspond to the models (a) CAM5.3, (b) ECHAM6.3-SALSA2.0, (c) ECHAM6.3-HAM2.3, (d) HadGEM3, (e) OsloCTM2, (f) GEOS-Chem, and (g) CAM5.4. Observations used in Figs. 1,2, and Supplementary Figure 3 are shown in panel h. The solid rectangles represent the different model regions isolated for biomass burning occurrence.

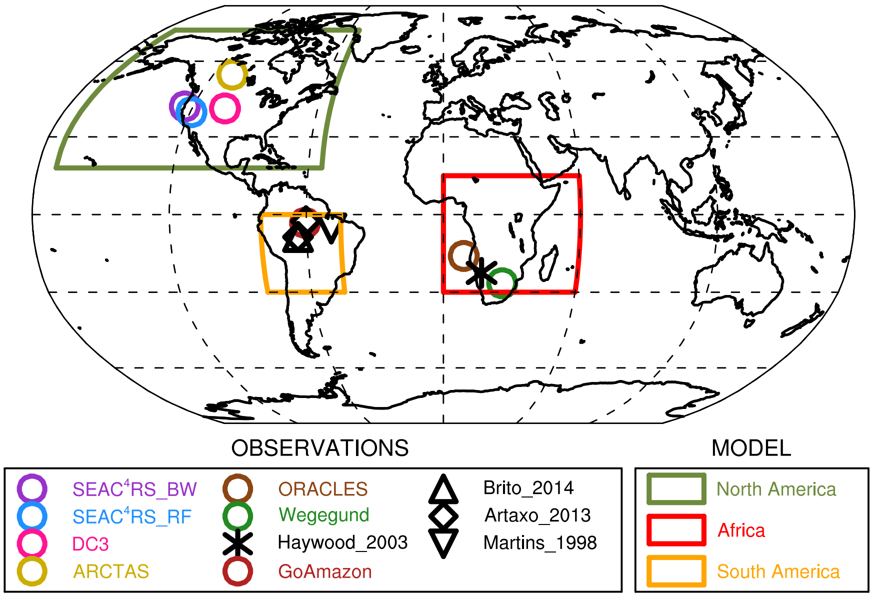

Supplementary Figure 2: Observational datasets and CAM5.4 regions used only in the comparison of modeled and observed size distributions (Supplementary Figure 3). Observations are represented by symbols and the model regions are designated by the solid boxes.

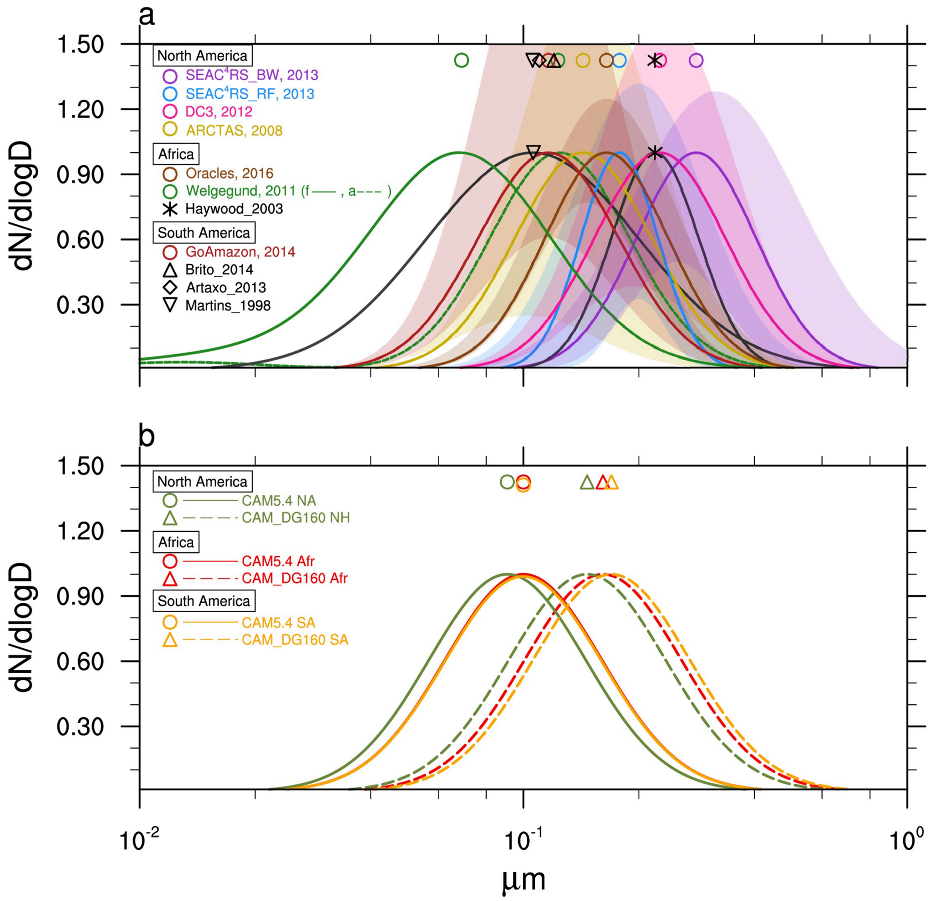

Supplementary Figure 3: Normalized aerosol size distributions from observations and CAM5.4 simulations. The panels describe (a) biomass burning observations and (b) primary carbon mode from CAM5.4 simulations. Color fill in panel (a) represents the range in the min and max number values reported in the observational datasets. The colors in panel (a) correspond to those in Fig. 1 and Supplementary Figure 1, with locations of the observations described in Supplementary Figure 2. Panel (b) describes two model simulations: CAM5.4 (circle; solid line) and CAM_DG160 (triangle; dashed line). Model regions are described in Supplementary Figure 2.

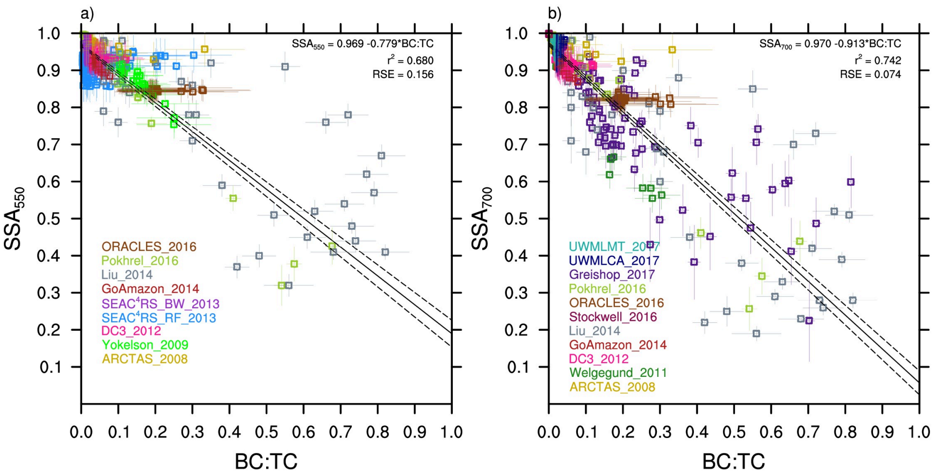

Supplementary Figure 4: Single scattering albedo (SSA) versus BC/(BC+OC) (i.e., BC:TC) for observations at 550 (a) and 700 (b) nm wavelengths. Same as Fig. 1, with expanded BC:TC and SSA ranges.

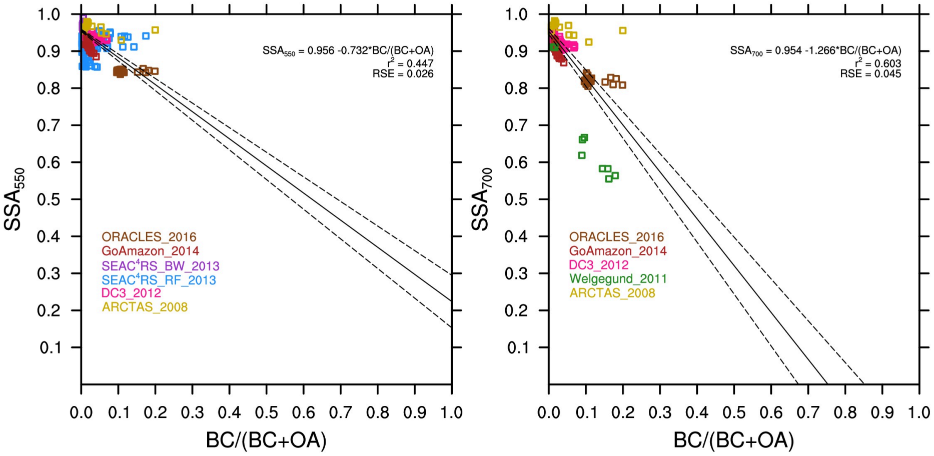

Supplementary Figure 5: Single scattering albedo (SSA) versus BC/(BC+OA) for observations at 550 (a) and 700 (b) nm wavelengths. Same as Supplementary Figure 4 but only using observations that contain information about OA concentrations.

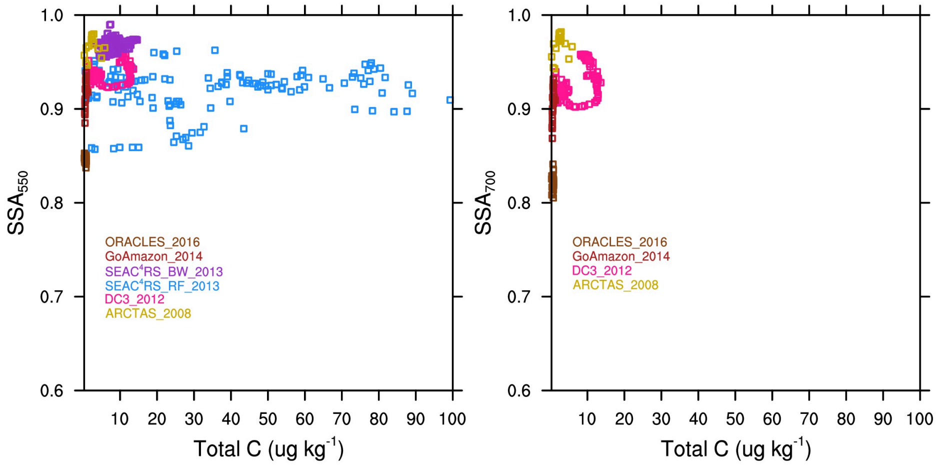

Supplementary Figure 6: Single scattering albedo (SSA) versus total carbon (Total C; BC+OC, µg kg^{-1}) for observations at 550 (a) and 700 (b) nm wavelengths. Observations were chosen based on availability of organic carbon mass mixing ratio.

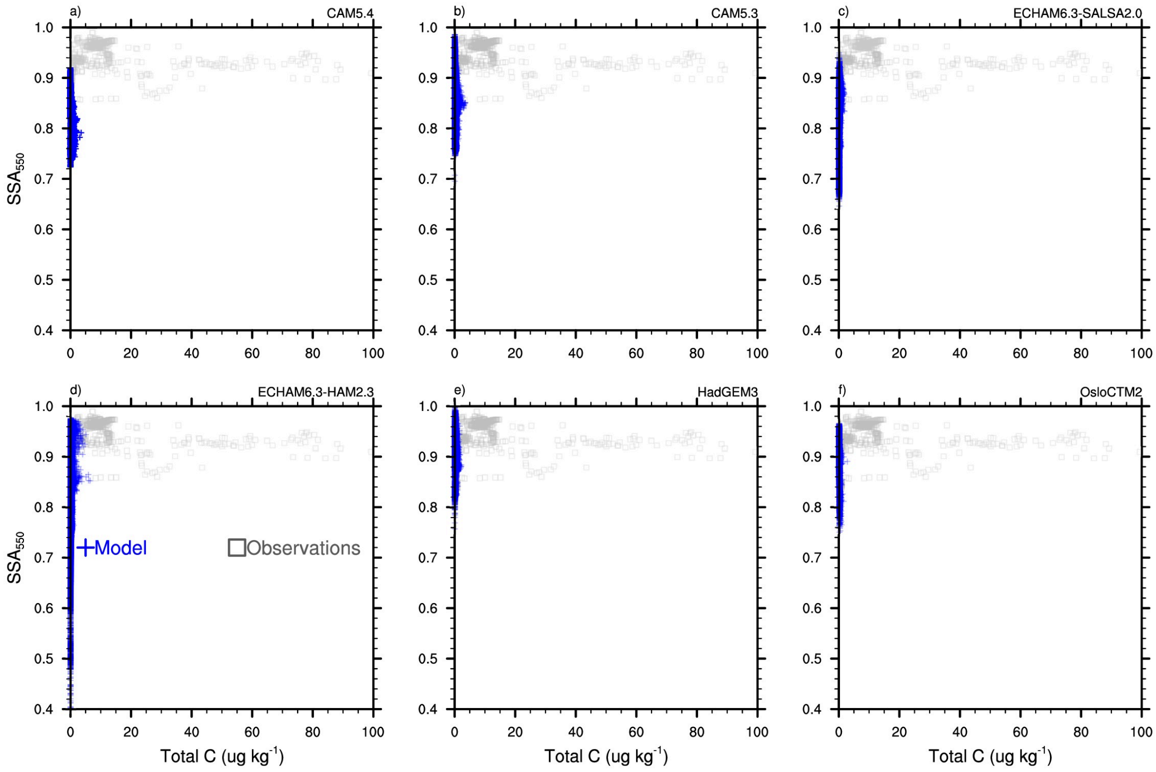

Supplementary Figure 7: Single scattering albedo (SSA) versus total carbon (Total C; BC+OC, µg kg^{-1}) at 550 (a) and 700 (b) nm wavelengths for the observations from Supplementary Figure 6, and 6 of the model simulations: (a) CAM5.4, (b) CAM5.3, (c) ECHAM6.3-SALSA2.0, (d) ECHAM6.3-HAM2.3, (e) HadGEM3, and (f) OsloCTM2.

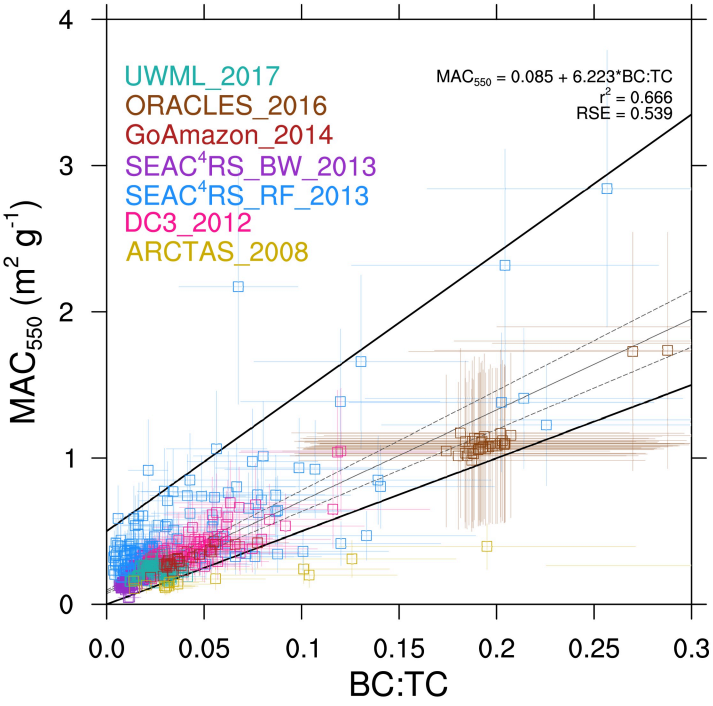

Supplementary Figure 8: Biomass burning (BB) mass absorption cross-section (MAC_{BB}; abs. coeff. / ([BC] + [OA]), m^2 g^{-1}) versus BC/(BC+OC) (i.e., BC:TC). Same as Fig. 3, but detailing the color-coded observational datasets used in the comparison.

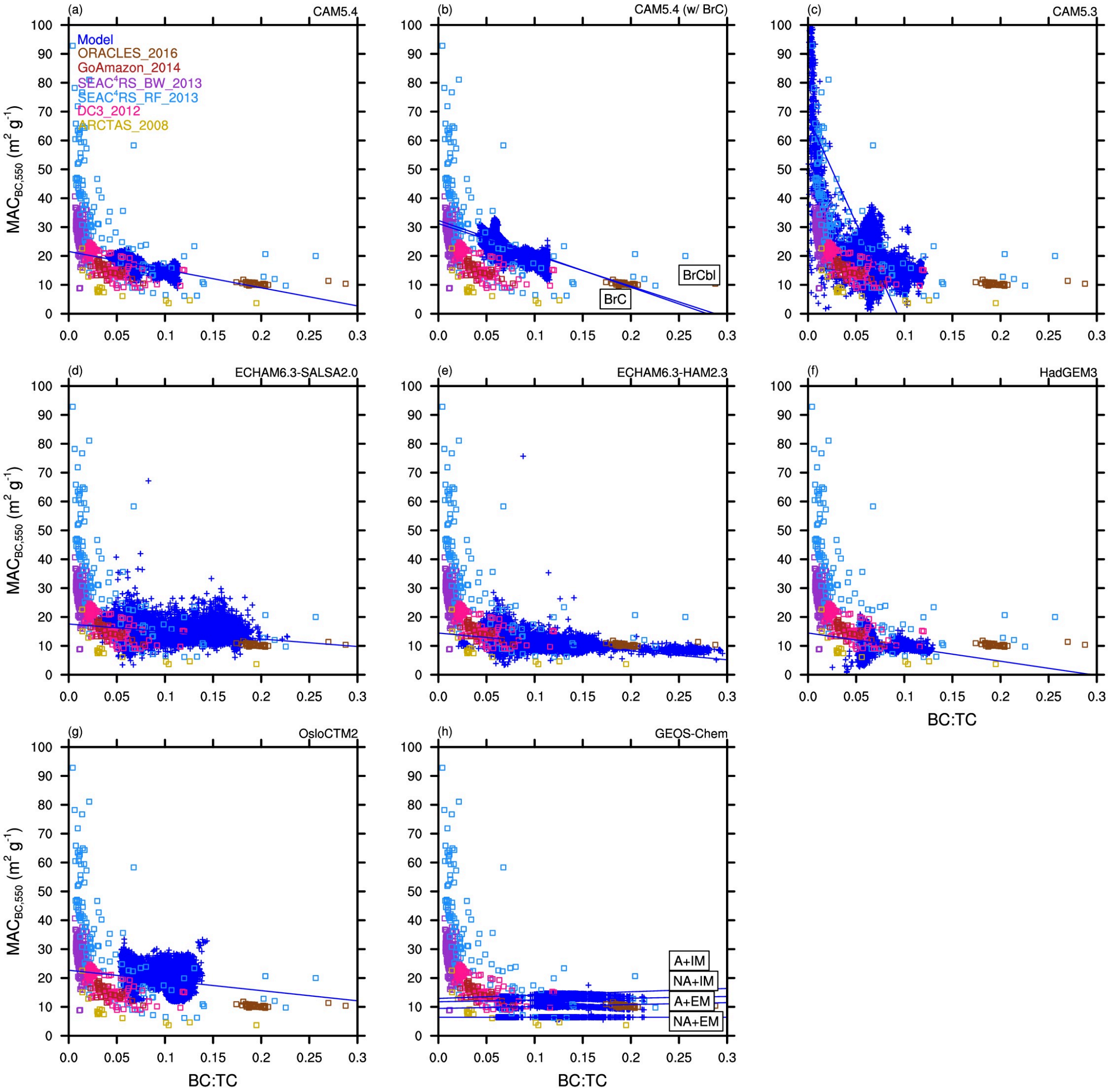

Supplementary Figure 9: Same as Fig. 3 but for black carbon (BC) mass absorption coefficient at 550 nm ($MAC_{BC,550}$; abs. coeff / ([BC]), m^2 g^{-1}) versus black carbon to total carbon ratio (BC:TC). High sensitivity in observational $MAC_{BC,550}$ at low BC:TC (< ~0.04) are attributed to overestimation of absorption coefficient by the Particle Soot Absorption Photometer (PSAP) due to multiple scattering issues[70], BC absorption enhancement, and contribution to absorption by light absorbing organic aerosol (i.e., brown carbon; see Supplementary Figure 10). High $MAC_{BC,550}$ in the CAM5.3 simulation is attributed to BC absorption enhancement at low BC mass concentrations (corresponding to upper level transport).

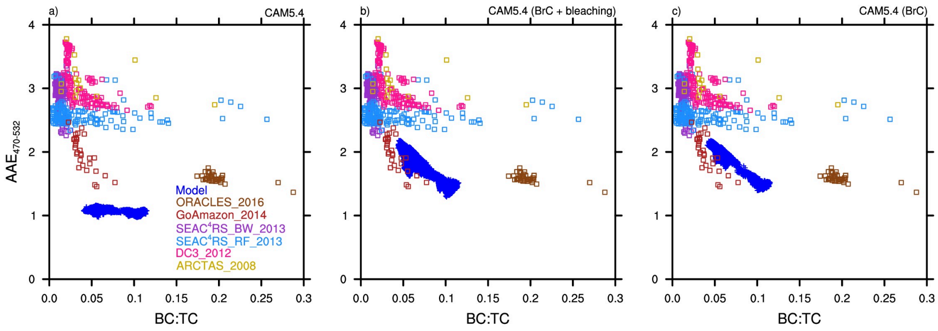

Supplementary Figure 10: Biomass burning absorption Angstrom exponent (AAE) for the wavelengths of the two lower Particle Soot Absorption Photometer (PSAP) absorption channels (470 nm to 532 nm) from observations and three CAM5.4 model simulations. These simulations are the default CAM5.4 simulations (a) without brown carbon, (b) with brown carbon and a photochemical bleaching effect, and (c) with brown carbon and no photochemical bleaching. Higher AAE are an indication of stronger wavelength dependence for the visible light absorption, which is also a characteristic of brown carbon[71]. Lack of wavelength dependence (AAE ~ 1) in the CAM5.4 model simulation in panel a) indicates a lack of brown carbon parameterization in the default model configuration (Brown et al.[5]).

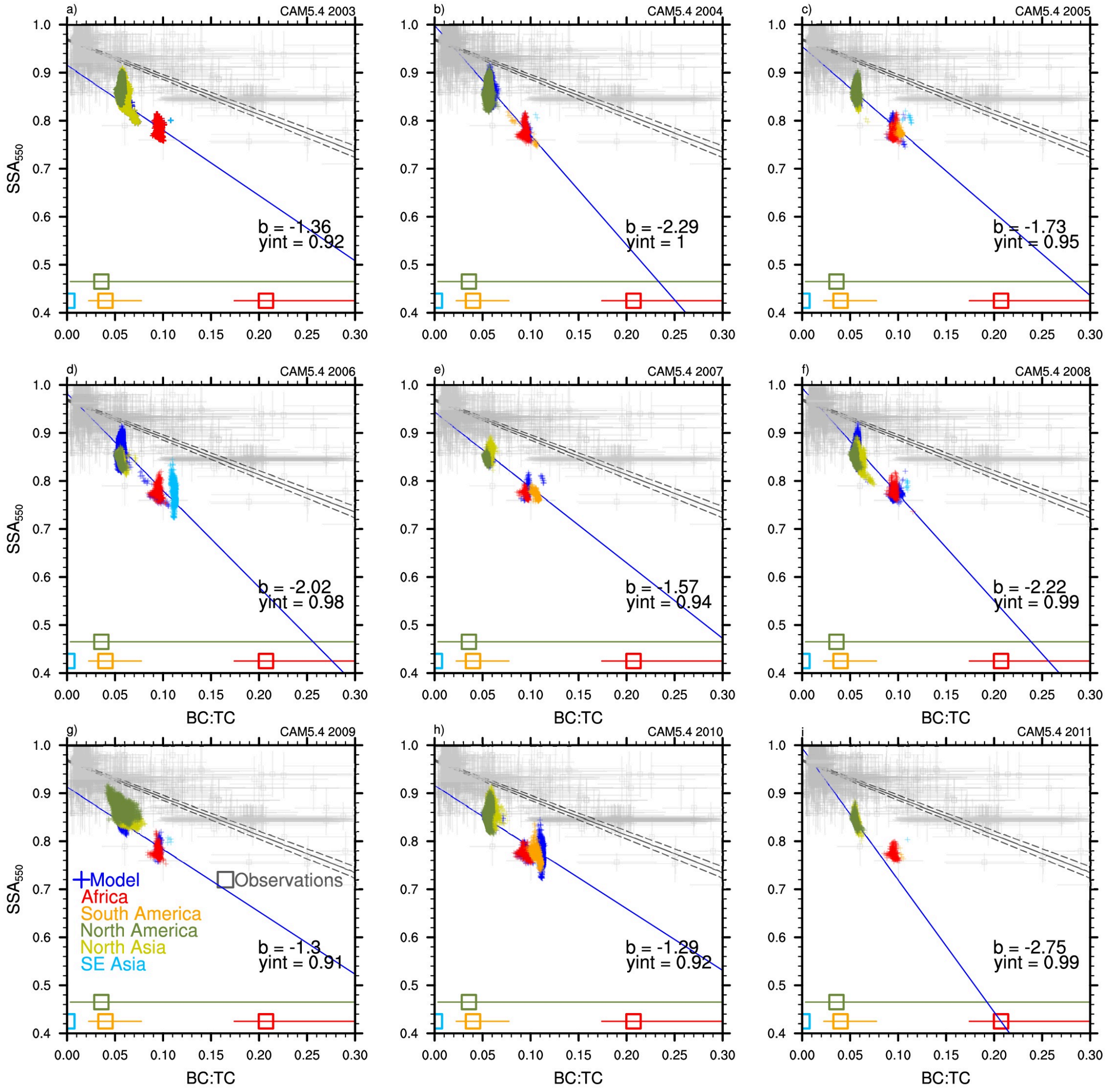

Supplementary Figure 11: Interannual comparison of CAM5.4 single scattering albedo (SSA) versus black carbon to total carbon ratio (BC:TC). The different panels show each of the nine years from the 2003-2011 CAM5.4 simulation. The slope and y-intercept of the linear fit (blue line) are included in each panel.

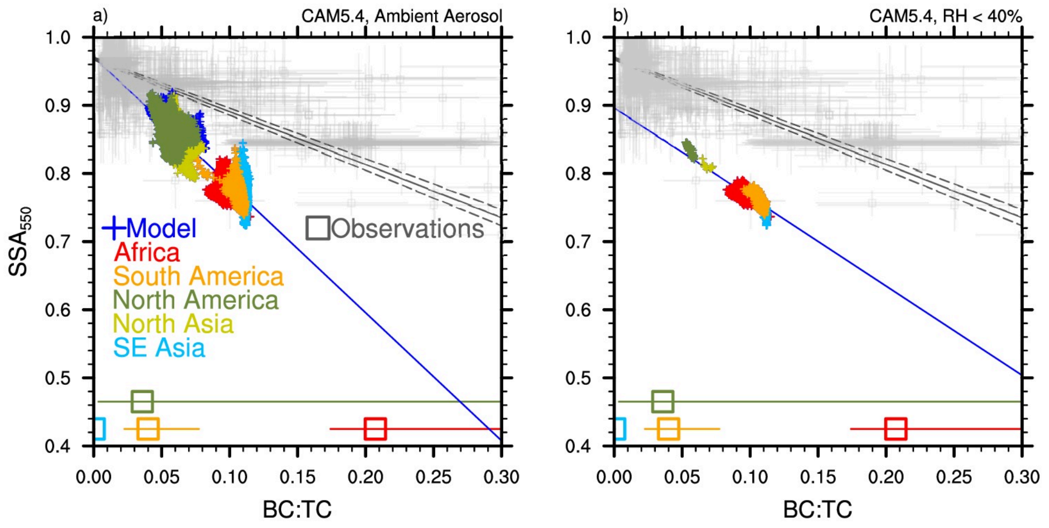

Supplementary Figure 12: Testing the effect of grid cell relative humidity on biomass burning (BB) single scattering albedo (SSA) versus black carbon to total carbon ratio (BC:TC) in CAM5.4. Panel (a) shows ambient aerosol conditions, while panel (b) shows an additional comparison with BB aerosols processed to only include aerosol at relative humidity less than 40%. This is based on ideal aerosol capture conditions outlined in WMO/GAW[72].

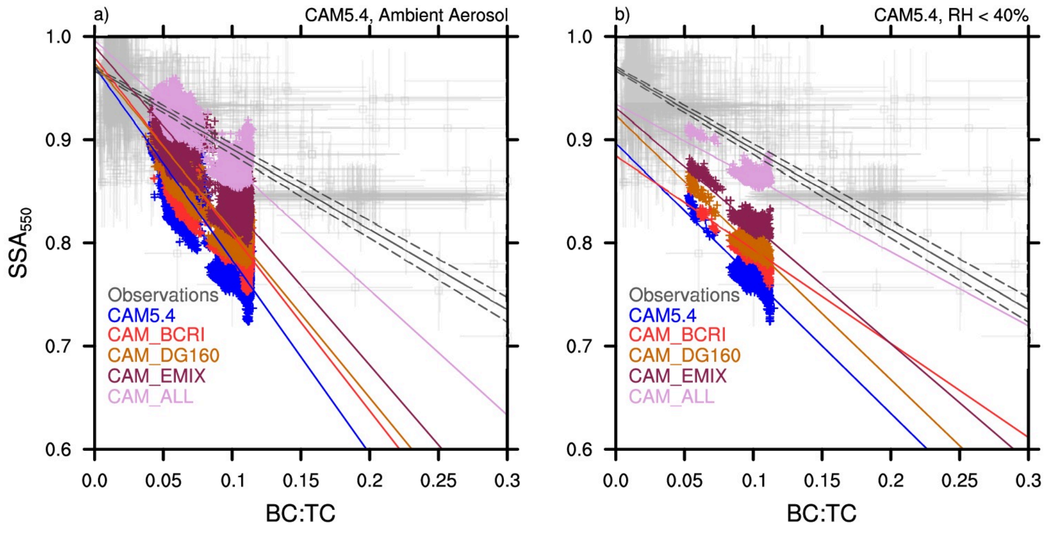

Supplementary Figure 13: Testing the effect of grid cell relative humidity on biomass burning (BB) single scattering albedo (SSA) versus black carbon to total carbon ratio (BC:TC) in CAM5.4 and the BB microphysics sensitivity simulations used in this study.

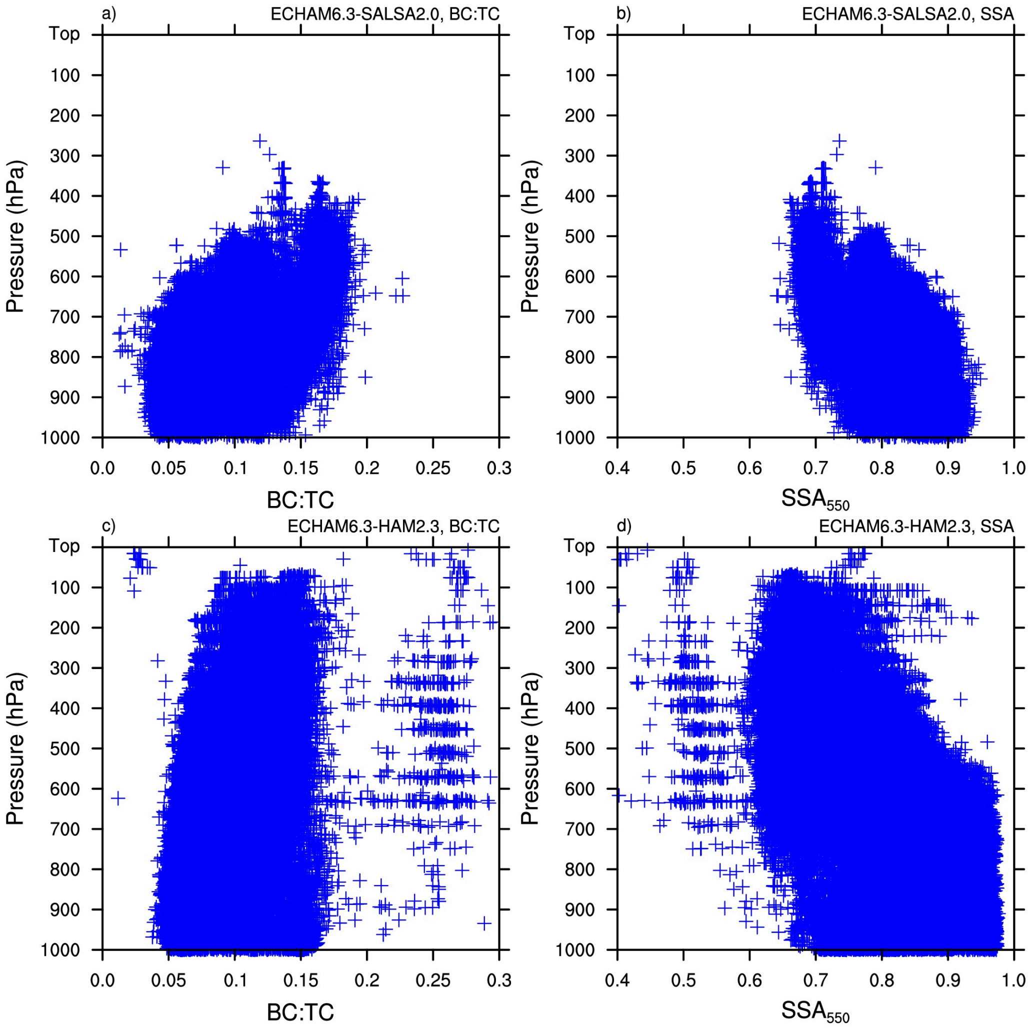

Supplementary Figure 14: Comparison of (a,c) model vertical level vs. black carbon to total carbon ratio (BC:TC) and (b,d) model vertical level vs. single scattering albedo (SSA) at 550 nm for both ECHAM6.3-SALSA2 (top row) and ECHAM6.3-HAM2.3 (bottom row).

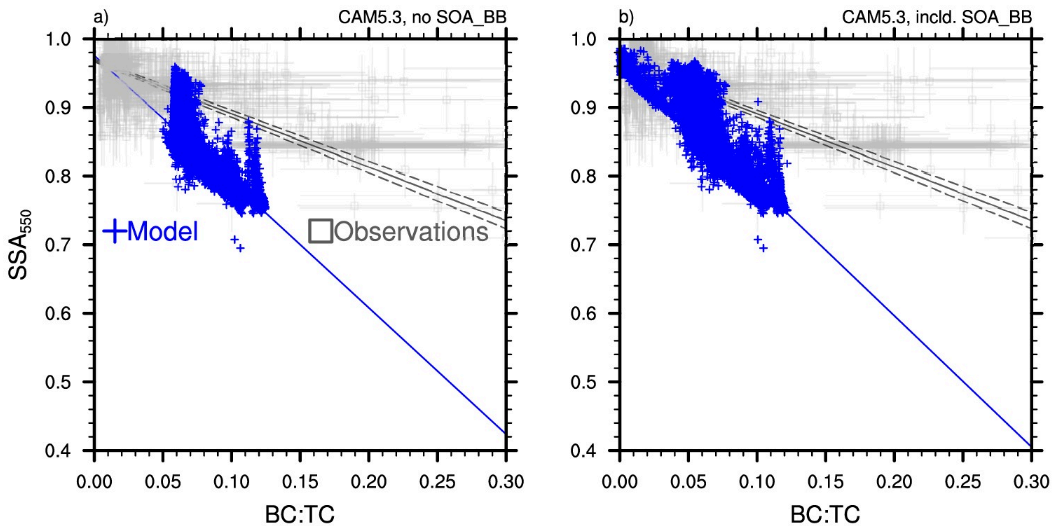

Supplementary Figure 15: Testing the effect of biomass burning (BB) secondary organic aerosol (SOA) in CAM5.3. Panels show CAM5.3 without (a) and with (b) SOA BB (panel (b) is identical to Fig. 2c). SOA BB is calculated by the following: SOA (simulation with BB aerosol) – SOA (simulation without BB aerosol). This describes the SOA that condenses on BB aerosol.

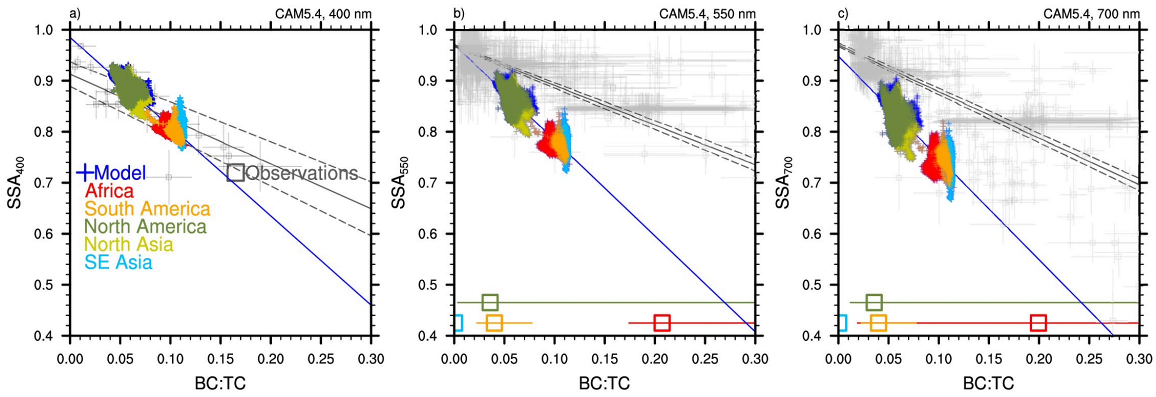

Supplementary Figure 16: Comparing single scattering albedo (SSA) versus black carbon to total carbon ratio (BC:TC) from CAM5.4 at three different wavelengths: (a) 400 nm, (b) 550 nm, and (c) 700 nm. Observations for panels (b) and (c) are the same as for Fig. 1, while observations for panel (a) are from Pokhrel et al.[55], which are part of observations in b) and c).

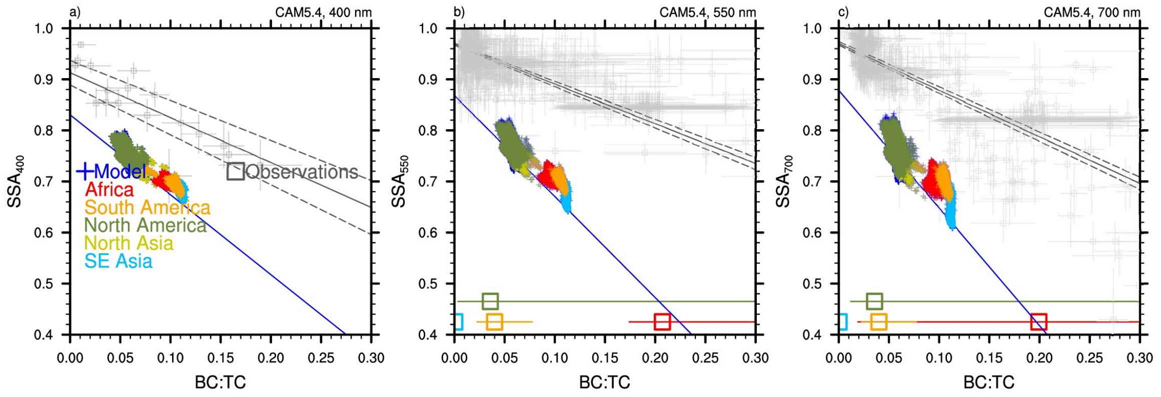

Supplementary Figure 17: Comparing single scattering albedo (SSA) versus black carbon to total carbon ratio (BC:TC) from CAM5.4 (w/ brown carbon) at three different wavelengths: (a) 400 nm, (b) 550 nm, and (c) 700 nm. Observations for panels (b) and (c) are the same as for Fig. 1, while observations for panel (a) are from Pokhrel et al.[55], which are part of observations in b) and c).

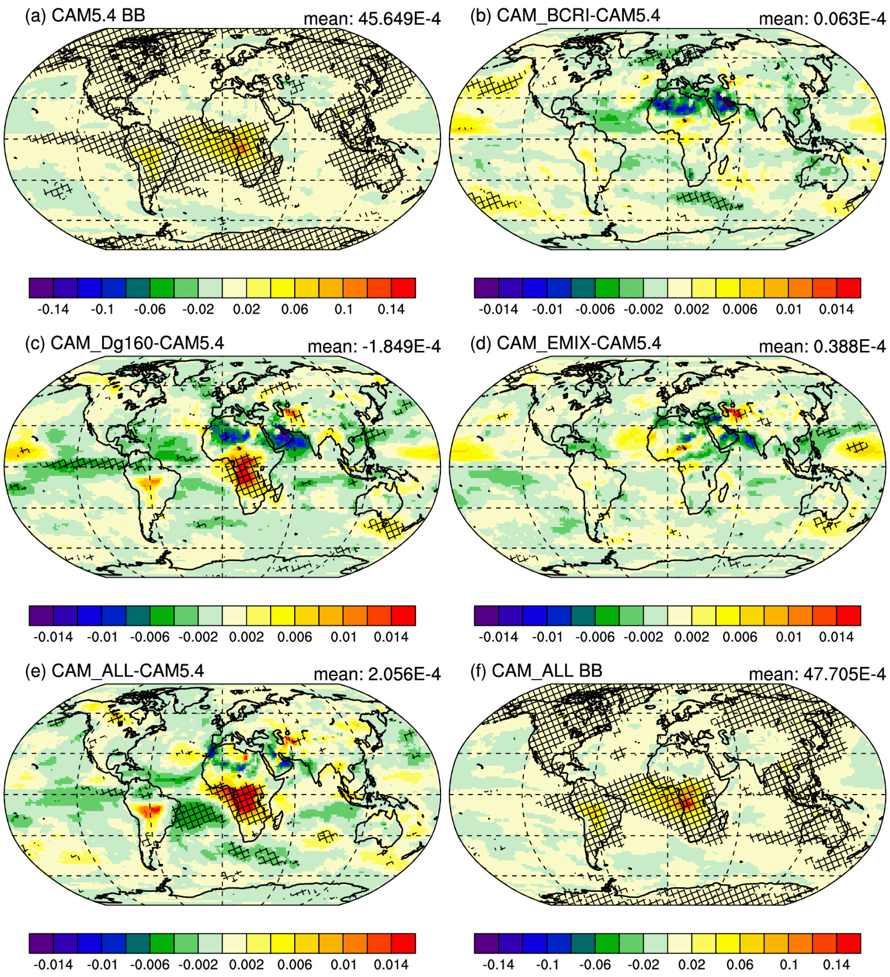

Supplementary Figure 18: Aerosol scattering optical depth (ASOD, aerosol optical depth (AOD) – absorption aerosol optical depth (AAOD)) of biomass burning aerosol (BB) in CAM5.4. The panels are (a) default CAM5.4 BB ASOD, (b) the difference in ASOD due to changes in BB black carbon refractive index (CAM_ BCRI – CAM5.4), (c) the difference in ASOD due to increasing BB aerosol size (CAM_Dg160 – CAM5.4), (d) the difference in ASOD due to treating fresh BB aerosol as externally mixed (CAM_EMIX – CAM5.4), (e) the difference in ASOD due to all of the previous changes (CAM_ALL – CAM5.4), and (f) the ASOD of BB with all of the previous changes (CAM_ALL). Hatching indicates regions where the change over the ensemble years is significant to the 0.05 level. Note difference in color bars.

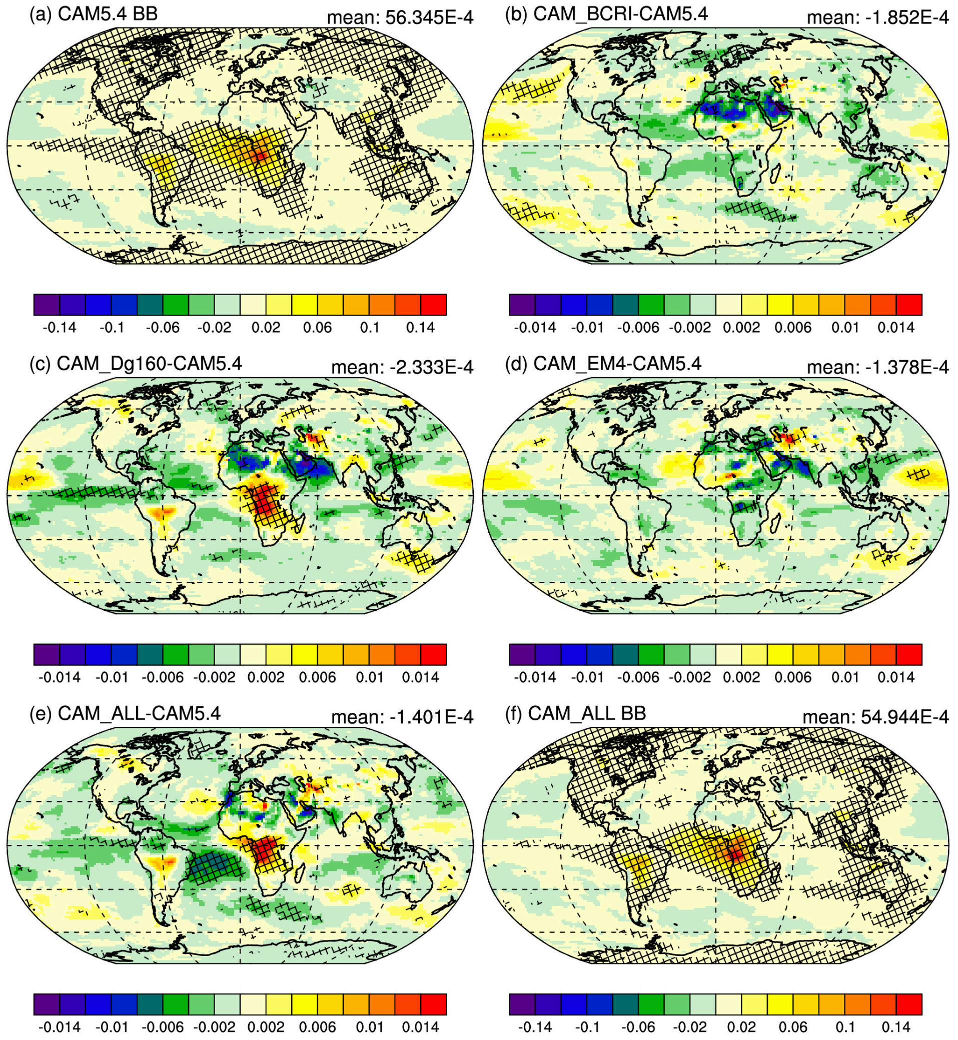

Supplementary Figure 19: Aerosol optical depth (AOD) of biomass burning aerosol (BB) in CAM5.4. The panels are (a) default CAM5.4 BB AOD, (b) the difference in AOD due to changes in BB black carbon refractive index (CAM_ BCRI – CAM5.4), (c) the difference in AOD due to increasing BB aerosol size (CAM_Dg160 – CAM5.4), (d) the difference in AOD due to treating fresh BB aerosol as externally mixed (CAM_EMIX – CAM5.4), (e) the difference in AOD due to all of the previous changes (CAM_ALL – CAM5.4), and (f) the AOD of BB with all of the previous changes (CAM_ALL). Hatching indicates regions where the change over the ensemble years is significant to the 0.05 level. Note difference in color bars.

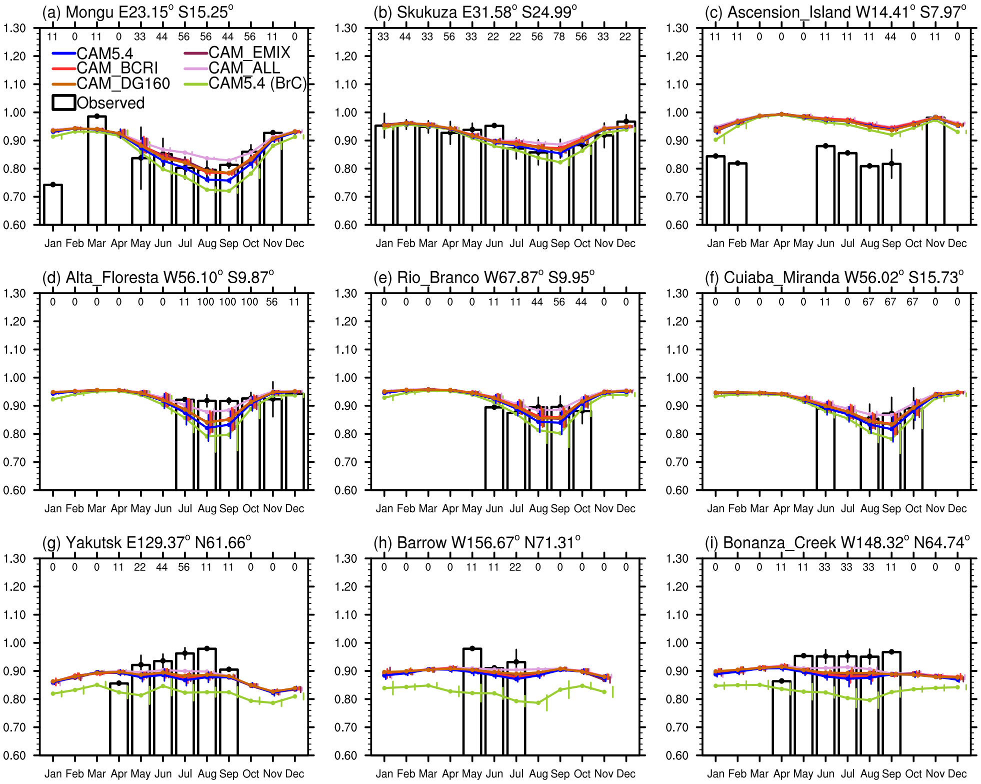

Supplementary Figure 20: Aerosol Robotic Network (AERONET) and model comparison of single scattering albedo (SSA) for the wavelengths of 675 nm for AERONET and 700 nm for CAM5.4. This comparison is the same as that in Brown et al.[5], and compares AERONET sites influenced by African (a-c), South American (d-f), and Arctic (g-i) biomass burning (BB) emissions (black bars) to model SSA from the same regions. The models are default CAM5.4 (CAM5.4; blue), CAM5.4 with decreased BB black carbon refractive index (CAM_BCRI; red), CAM5.4 with increased BB aerosol size (CAM_Dg160; gold), CAM5.4 with externally mixed, fresh BB aerosol (CAM_EMIX; maroon), CAM5.4 with all of the previous changes (CAM_ALL; pink), and CAM5.4 with brown carbon (CAM (BrC); green). Vertical lines are observation and model standard devations and run from left to right as follows: Observations (black), CAM5.4 (blue), CAM_BCRI (red), CAM_Dg160 (gold), CAM_EMIX (maroon), CAM_ALL (pink), CAM (BrC) (green). Values below the upper x axis indicate percentage of available data in the 9-year period.

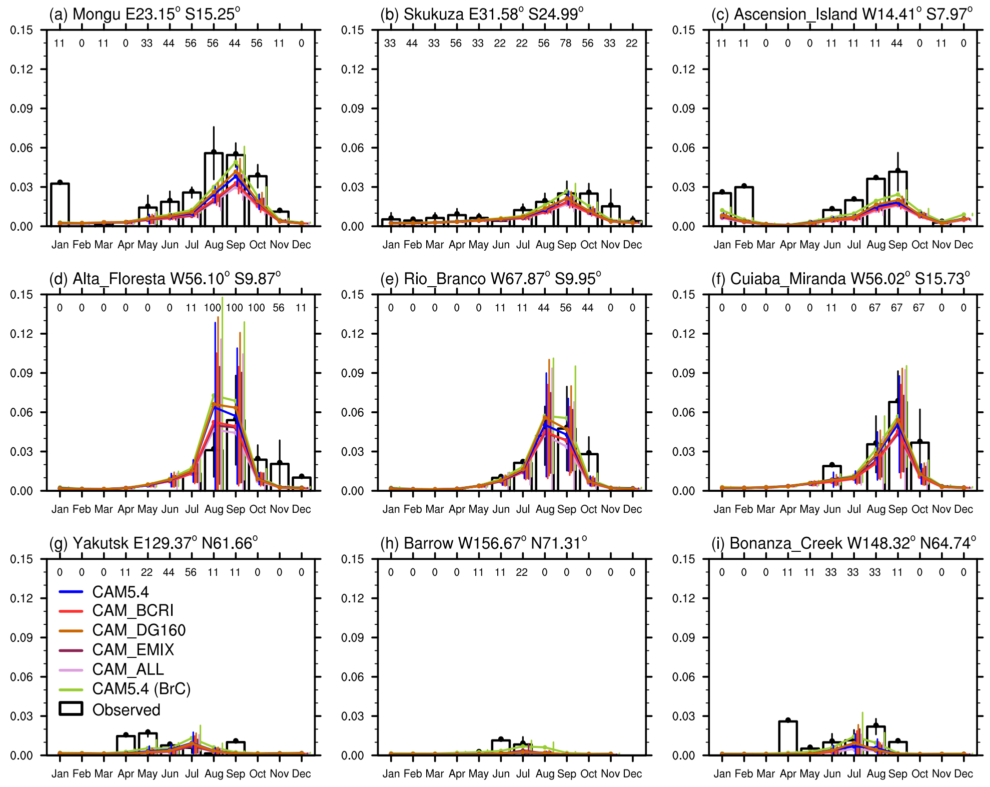

Supplementary Figure 21: Aerosol Robotic Network (AERONET) and model comparison of aerosol absorption optical depth (AAOD) for the wavelengths of 675 nm for AERONET and 700 nm for CAM5.4. This comparison is the same as that in Brown et al.[5], and compares AERONET sites influenced by African (a-c), South American (d-f), and Arctic (g-i) biomass burning (BB) emissions (black bars) to model AAOD from the same regions. The models are default CAM5.4 (CAM5.4; blue), CAM5.4 with decreased BB black carbon refractive index (CAM_BCRI; red), CAM5.4 with increased BB aerosol size (CAM_Dg160; gold), CAM5.4 with externally mixed, fresh BB aerosol (CAM_EMIX; maroon), CAM5.4 with all of the previous changes (CAM_ALL; pink), and CAM5.4 with brown carbon (CAM (BrC); green). Vertical lines are observation and model standard devations and run from left to right as follows: Observations (black), CAM5.4 (blue), CAM_BCRI (red), CAM_Dg160 (gold), CAM_EMIX (maroon), CAM_ALL (pink), CAM (BrC) (green). Values below the upper x axis indicate percentage of available data in the 9-year period.

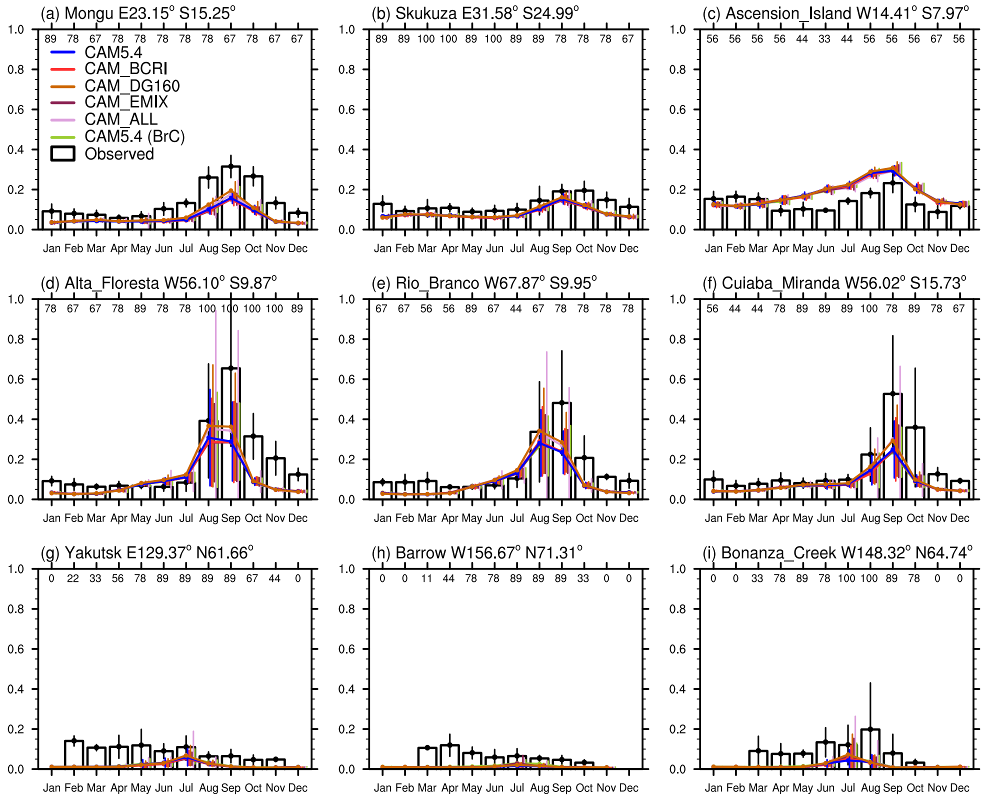

Supplementary Figure 22: Aerosol Robotic Network (AERONET) and model comparison of aerosol optical depth (AOD) for the wavelengths of 675 nm for AERONET and 700 nm for CAM5.4. This comparison is the same as that in Brown et al.[5], and compares AERONET sites influenced by African (a-c), South American (d-f), and Arctic (g-i) biomass burning (BB) emissions (black bars) to model AOD from the same regions. The models are default CAM5.4 (CAM5.4; blue), CAM5.4 with decreased BB black carbon refractive index (CAM_BCRI; red), CAM5.4 with increased BB aerosol size (CAM_Dg160; gold), CAM5.4 with externally mixed, fresh BB aerosol (CAM_EMIX; maroon), CAM5.4 with all of the previous changes (CAM_ALL; pink), and CAM5.4 with brown carbon (CAM (BrC); green). Vertical lines are observation and model standard devations and run from left to right as follows: Observations (black), CAM5.4 (blue), CAM_BCRI (red), CAM_Dg160 (gold), CAM_EMIX (maroon), CAM_ALL (pink), CAM (BrC) (green). Values below the upper x axis indicate percentage of available data in the 9-year period.

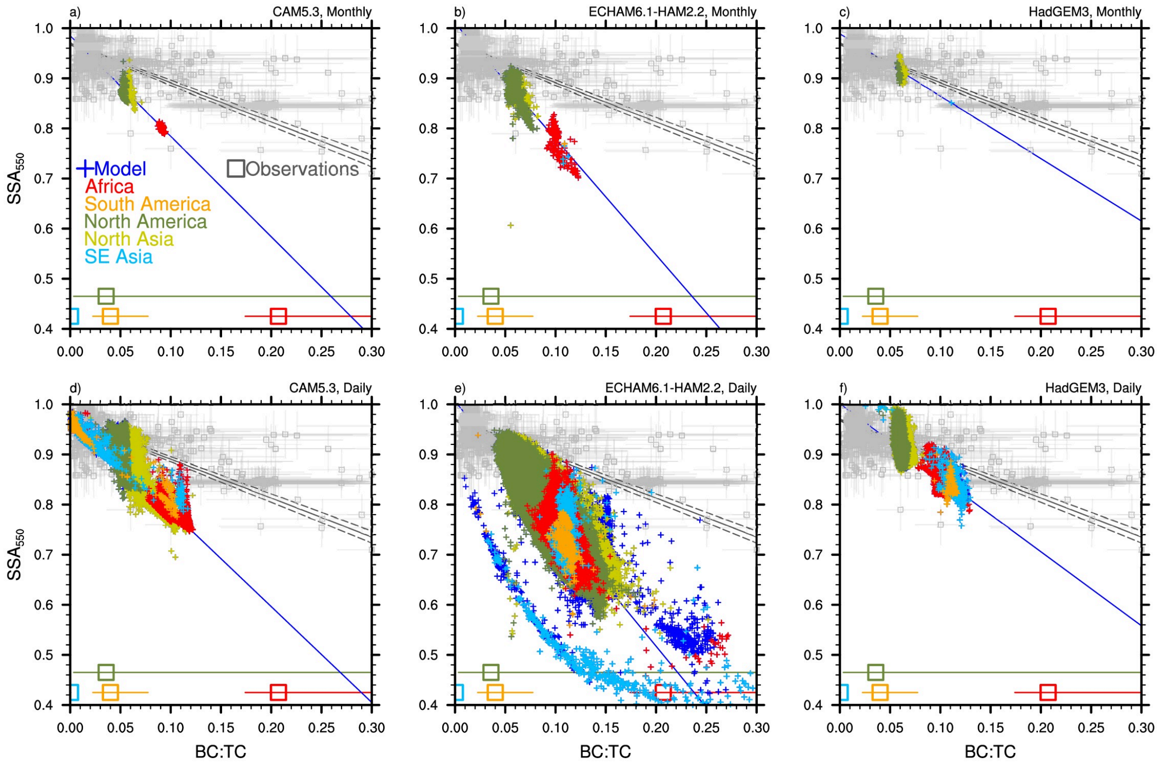

Supplementary Figure 23: Comparison between single scattering albedo (SSA) and black carbon to total carbon ratio (BC:TC) for monthly and daily model output. Monthly (upper row) and daily (bottom row) temporal resolutions are reported from the models CAM5.3 (a,d), ECHAM6.1-HAM2.2 (b,e), and HadGEM3 (c,f). Only three models are presented in this comparison as these are the only simulations with both monthly and daily output.

Supplementary references:

1. Liu, X. *et al.* Toward a minimal representation of aerosols in climate models: description and evaluation in the Community Atmosphere Model CAM5. *Geoscientific Model Development* **5**, 709–739 (2012).
2. Bretherton, C. S. & Park, S. A New Moist Turbulence Parameterization in the Community Atmosphere Model. *J. Climate* **22**, 3422–3448 (2009).
3. Liu, X. *et al.* Description and evaluation of a new four-mode version of the Modal Aerosol Module (MAM4) within version 5.3 of the Community Atmosphere Model. *Geoscientific Model Development* **9**, 505–522 (2016).
4. Neale, R. B. *et al.* : Description of the NCAR Community Atmosphere Model (CAM5.0), Tech. Rep. NCAR/TN-486+STR, National Center for Atmospheric Research, Boulder, CO, USA (2012).
5. Brown, H. *et al.* Radiative effect and climate impacts of brown carbon with the Community Atmosphere Model (CAM5). *Atmospheric Chemistry and Physics* **18**, 17745–17768 (2018).
6. Bergman, T. *et al.* Evaluation of the sectional aerosol microphysics module SALSA implementation in ECHAM5-HAM aerosol-climate model. *Geosci. Model Dev.* **5**, 845–868 (2012).
7. Stier, P. *et al.* The aerosol-climate model ECHAM5-HAM. *Atmos. Chem. Phys.* 32 (2005).
8. Stevens, B. *et al.* Atmospheric component of the MPI-M Earth System Model: ECHAM6: ECHAM6. *J. Adv. Model. Earth Syst.* **5**, 146–172 (2013).
9. Kokkola, H. *et al.* SALSA2.0: The sectional aerosol module of the aerosol–chemistry–climate model ECHAM6.3.0-HAM2.3-MOZ1.0. *Geosci. Model Dev.* **11**, 3833–3863 (2018).
10. Laakso, A. *et al.* Radiative and climate impacts of a large volcanic eruption during stratospheric sulfur geoengineering. *Atmos. Chem. Phys.* **16**, 305–323 (2016).
11. Croft, B. *et al.* Aerosol size-dependent below-cloud scavenging by rain and snow in the ECHAM5-HAM. *Atmos. Chem. Phys.* **9**, 4653–4675 (2009).
12. Croft, B. *et al.* Influences of in-cloud aerosol scavenging parameterizations on aerosol concentrations and wet deposition in ECHAM5-HAM. *Atmos. Chem. Phys.* **10**, 1511–1543 (2010).
13. Zhang, K. *et al.* The global aerosol-climate model ECHAM-HAM, version 2: sensitivity to improvements in process representations. *Atmos. Chem. Phys.* **12**, 8911–8949 (2012).
14. Seinfeld, J. H. and Pandis, S. N. Atmospheric Chemistry and Physics: From Air Pollution to Climate Change, 1st edition. *J. Wiley*, New York. (1998).
15. Slinn, S. and Slinn, W. Predictions for particle deposition on natural waters, *Atmos. Environ*. **14**, 1013–1016 (1980).
16. Val Martin, M. *et al.* Smoke injection heights from fires in North America: analysis of 5 years of satellite observations. *Atmos. Chem. Phys.* **10**, 1491–1510 (2010).

17. Tegen, I. *et al.* The global aerosol–climate model ECHAM6.3–HAM2.3 – Part 1: Aerosol evaluation. *Geosci. Model Dev.* **12**, 1643–1677 (2019).
18. Wang, Q. *et al.* Sources of carbonaceous aerosols and deposited black carbon in the Arctic in winter-spring: implications for radiative forcing. *Atmos. Chem. Phys.* **11**, 12453–12473 (2011).
19. Liu, H., Jacob, D. J., Bey, I. & Yantosca, R. M. Constraints from 210Pb and 7Be on wet deposition and transport in a global three-dimensional chemical tracer model driven by assimilated meteorological fields. *J. Geophys. Res.* **106**, 12109–12128 (2001).
20. Fairlie, T. D., Jacob, D. J. & Park, R. J. The impact of transpacific transport of mineral dust in the United States. *Atmospheric Environment* **41**, 1251–1266 (2007).
21. Alexander, B. Sulfate formation in sea-salt aerosols: Constraints from oxygen isotopes. *J. Geophys. Res.* **110**, D10307 (2005).
22. Lin, J.-T., Wuebbles, D. J. & Liang, X.-Z. Effects of intercontinental transport on surface ozone over the United States: Present and future assessment with a global model. *Geophys. Res. Lett.* **35**, L02805 (2008).
23. Bey, I. *et al.* Global modeling of tropospheric chemistry with assimilated meteorology: Model description and evaluation. *J. Geophys. Res.* **106**, 23073–23095 (2001).
24. Saleh, R. *et al.* Contribution of brown carbon and lensing to the direct radiative effect of carbonaceous aerosols from biomass and biofuel burning emissions. *Journal of Geophysical Research: Atmospheres.* (2015).
25. Mann, G. W. *et al.* Description and evaluation of GLOMAP-mode: a modal global aerosol microphysics model for the UKCA composition-climate model. *Geosci. Model Dev.* **3**, 519–551 (2010).
26. Bellouin, N. *et al.* Impact of the modal aerosol scheme GLOMAP-mode on aerosol forcing in the Hadley Centre Global Environmental Model. *Atmos. Chem. Phys.* **13**, 3027–3044 (2013).
27. Johnson, B. T. *et al.* Evaluation of biomass burning aerosols in the HadGEM3 climate model with observations from the SAMBBA field campaign. *Atmospheric Chemistry and Physics* **16**, 14657–14685 (2016).
28. Schulz, M. *et al.* Emission data sets and methodologies for estimating emissions. (2007).
29. Skeie, R. B. *et al.* Anthropogenic radiative forcing time series from pre-industrial times until 2010. *Atmospheric Chemistry and Physics* **11**, 11827–11857 (2011).
30. Myhre, G. *et al.* Modelled radiative forcing of the direct aerosol effect with multi-observation evaluation. *Atmospheric Chemistry and Physics* **9**, 1365–1392 (2009).
31. Bond, T. C. & Bergstrom, R. W. Light Absorption by Carbonaceous Particles: An Investigative Review. *Aerosol Science and Technology* **40**, 27–67 (2006).
32. Saleh, R. *et al.* Brownness of organics in aerosols from biomass burning linked to their black carbon content. *Nature Geoscience* **7**, 647–650 (2014).
33. Wang, X. *et al.* Exploring the observational constraints on the simulation of brown carbon. *Atmos. Chem. Phys.* **18**, 635–653 (2018).

34. Jiang, Y. *et al.* Impacts of global wildfire aerosols on direct radiative, cloud and surface-albedo forcings simulated with CAM5. *Atmospheric Chemistry and Physics Discussions* 1–54 (2016).
35. Jacobson, M. Z. Fundamentals of Atmospheric Modeling, 2nd Edn., Cambridge University Press, New York (2005).
36. Turpin, B. J. & Lim, H.-J. Species Contributions to PM2.5 Mass Concentrations: Revisiting Common Assumptions for Estimating Organic Mass. *Aerosol Science and Technology* **35**, 602–610 (2001).
37. Reddington, C. L. *et al*. The mass and number size distributions of black carbon aerosol over Europe. *Atmos. Chem. Phys.* **13**, 4917–4939 (2013).
38. Lin, G. *et al.* Radiative forcing of organic aerosol in the atmosphere and on snow: Effects of SOA and brown carbon. *Journal of Geophysical Research: Atmospheres* **119**, 7453–7476 (2014).
39. World Climate Programme (WCP): Report of the experts meeting on aerosols and their climatic effects, edited by: Deepak, A. and Gerber, H. G., World Meteorological Organization, Geneva, Switzerland, Rep. WCP-55, 107 pp., (1983).
40. Haywood, J. M. *et al.* The mean physical and optical properties of regional haze dominated by biomass burning aerosol measured from the C-130 aircraft during SAFARI 2000. *J. Geophys. Res.* **108**, 8473 (2003).
41. Formenti, P. *et al.* Inorganic and carbonaceous aerosols during the Southern African Regional Science Initiative (SAFARI 2000) experiment: Chemical characteristics, physical properties, and emission data for smoke from African biomass burning. *J. Geophys. Res.* **108**, n/a-n/a (2003).
42. Myhre, G. *et al.* Comparison of the radiative properties and direct radiative effect of aerosols from a global aerosol model and remote sensing data over ocean. *Tellus B* **59**, 115–129 (2007).
43. Magi, B. I. & Hobbs, P. V. Effects of humidity on aerosols in southern Africa during the biomass burning season. *Journal of Geophysical Research: Atmospheres* **108**, (2003).
44. Jacob, D. J. *et al.* The Arctic Research of the Composition of the Troposphere from Aircraft and Satellites (ARCTAS) mission: design, execution, and first results. *Atmospheric Chemistry and Physics* **10**, 5191–5212 (2010).
45. Artaxo, P. *et al.* Atmospheric aerosols in Amazonia and land use change: from natural biogenic to biomass burning conditions. *Faraday Discussions* **165**, 203–235 (2013).
46. Brito, J. *et al.* Ground-based aerosol characterization during the South American Biomass Burning Analysis (SAMBBA) field experiment. *Atmospheric Chemistry and Physics* **14**, 12069–12083 (2014).
47. Barth, M. C. *et al.* The Deep Convective Clouds and Chemistry (DC3) Field Campaign. *Bull. Amer. Meteor. Soc.* **96**, 1281–1309 (2014).
48. Martin, S. T. *et al.* Introduction: Observations and Modeling of the Green Ocean Amazon (GoAmazon2014/5). *Atmospheric Chemistry and Physics* **16**, 4785–4797 (2016).
49. de Sá, S. S. *et al.* Contributions of biomass-burning, urban, and biogenic emissions tothe concentrations and light-absorbing properties of particulate matter

in central Amazonia during the dry season. *Atmos. Chem. Phys. Discuss.* 1–77 (2019) doi:10.5194/acp-2018-1309.
50. Grieshop, A. P., Jain, G., Sethuraman, K. & Marshall, J. D. Emission factors of health- and climate-relevant pollutants measured in home during a carbon-finance-approved cookstove intervention in rural India. *GeoHealth* **1**, 2017GH000066 (2017).
51. Liu, S. *et al.* Aerosol single scattering albedo dependence on biomass combustion efficiency: Laboratory and field studies. *Geophysical Research Letters* **41**, 742–748 (2014).
52. Martins, J. V. *et al.* Effects of black carbon content, particle size, and mixing on light absorption by aerosols from biomass burning in Brazil. *Journal of Geophysical Research: Atmospheres* **103**, 32041–32050 (1998).
53. Hobbs, P. V., Summary of types of data collected on the University of Washington's Convair C-131A aircraft in the Smoke, Clouds and Radiation-Brazil (SCAR-B) field study from 17 August-20 September 1995, Cloud and Aerosol Res. Group, Dep. of Atmos. Sci., Univ. of Washington, Seattle, March 1996.
54. Zuidema, P. *et al.* Smoke and Clouds above the Southeast Atlantic: Upcoming Field Campaigns Probe Absorbing Aerosol's Impact on Climate. *Bull. Amer. Meteor. Soc.* **97**, 1131–1135 (2016).
55. Pokhrel, R. P. *et al.* Parameterization of Single Scattering Albedo (SSA) and Absorption Angstrom Exponent (AAE) with EC/OC for Aerosol Emissions from Biomass Burning. *Atmospheric Chemistry and Physics Discussions* 1–27 (2016) doi:10.5194/acp-2016-184.
56. Toon, O. B. *et al.* Planning, implementation, and scientific goals of the Studies of Emissions and Atmospheric Composition, Clouds and Climate Coupling by Regional Surveys (SEAC4RS) field mission. *Journal of Geophysical Research: Atmospheres* **121**, 4967–5009 (2016).
57. Mason, B. *et al.* An intercomparison of aerosol absorption measurements conducted during the SEAC4RS campaign. *Aerosol Science and Technology* **52**, 1012–1027 (2018).
58. Stockwell, C. E. *et al.* Field measurements of trace gases and aerosols emitted by peat fires in Central Kalimantan, Indonesia, during the 2015 El Niño. *Atmos. Chem. Phys.* **16**, 11711–11732 (2016).
59. Foster, K., Pokhrel, R., Burkhart, M. & Murphy, S. A novel approach to calibrating a photoacoustic absorption spectrometer using polydisperse absorbing aerosol. *Atmos. Meas. Tech.* **12**, 3351–3363 (2019).
60. Vakkari, V. *et al.* Rapid changes in biomass burning aerosols by atmospheric oxidation. *Geophysical Research Letters* **41**, 2644–2651 (2014).
61. Vakkari, V. *et al.* Major secondary aerosol formation in southern African open biomass burning plumes. *Nature Geoscience* **11**, 580–583 (2018).
62. Yokelson, R. J. *et al.* Emissions from biomass burning in the Yucatan. *Atmospheric Chemistry and Physics* **9**, 5785–5812 (2009).
63. Bond, T. C., Anderson, T. L. & Campbell, D. Calibration and Intercomparison of Filter-Based Measurements of Visible Light Absorption by Aerosols. *Aerosol Science and Technology* **30**, 582–600 (1999).

64. Virkkula, A. Correction of the Calibration of the 3-wavelength Particle Soot Absorption Photometer (3λ PSAP). *Aerosol Science and Technology* **44**, 706–712 (2010).
65. Anderson, T. L. *et al*. Aerosol backscatter fraction and single scattering albedo: Measured values and uncertainties at a coastal station in the Pacific Northwest. *J. Geophys. Res*. **104**, 26793–26807 (1999).
66. Aiken, A. C. *et al.* O/C and OM/OC Ratios of Primary, Secondary, and Ambient Organic Aerosols with High-Resolution Time-of-Flight Aerosol Mass Spectrometry. *Environmental Science & Technology* **42**, 4478–4485 (2008).
67. Petzold, A. *et al.* Evaluation of Multiangle Absorption Photometry for Measuring Aerosol Light Absorption. *Aerosol Science and Technology* **39**, 40–51 (2005).
68. Tiitta, P. *et al.* Chemical composition, main sources and temporal variability of PM_1 aerosols in southern African grassland. *Atmospheric Chemistry and Physics* **14**, 1909–1927 (2014).
69. Müller, T., Laborde, M., Kassell, G. & Wiedensohler, A. Design and performance of a three-wavelength LED-based total scatter and backscatter integrating nephelometer. *Atmos. Meas. Tech.* **4**, 1291–1303 (2011).
70. Lack, D. A. *et al.* Bias in Filter-Based Aerosol Light Absorption Measurements Due to Organic Aerosol Loading: Evidence from Ambient Measurements. *Aerosol Science and Technology* **42**, 1033–1041 (2008).
71. Kirchstetter, T. W., Novakov, T. & Hobbs, P. V. Evidence that the spectral dependence of light absorption by aerosols is affected by organic carbon. *Journal of Geophysical Research: Atmospheres* **109**, n/a-n/a (2004).
72. WMO/GAW. Aerosol Measurement Procedures, Guidelines and Recommendation, Report No. 227. World Meteorological Organization, Geneva, Switzerland (2016).
